# Targeting c-Jun orchestrates heat stroke-induced myocardial injury and reveals its biomarker potential

**DOI:** 10.3389/fimmu.2025.1679750

**Published:** 2025-11-12

**Authors:** Yunfei Xiang, Rui Huang, Xuemei Jiang, Qingsong Chen, Can Luo, Hongxia Wang, Junyu Jiang, Jia Xie, Guangbin Huang, Menglong Liu, Yu Ma, Dingyuan Du, Fating Zhou

**Affiliations:** 1 Department of Traumatology, Chongqing Emergency Medical Center, Chongqing University Central Hospital, School of Medicine, Chongqing University, Chongqing, China; 2 Chongqing Key Laboratory of Emergency Medicine, Chongqing Emergency Medical Center, Chongqing University Central Hospital, School of Medicine, Chongqing University, Chongqing, China; 3 Department of Emergency Medicine, Chongqing Emergency Medical Center, Chongqing University Central Hospital, Chongqing, China; 4 Department of Emergency, Affiliated Hospital of Zunyi Medical University Zunyi, Guizhou, China; 5 Department of Traumatology, Chongqing Emergency Medical Center, Chongqing University Central Hospital, Chongqing, China; 6 Department of General Practice, People’s Hospital of Deyang City, Deyang, Sichuan, China

**Keywords:** heat stroke, myocardial injury, c-Jun, ZG-10, JNK/p38MAPK signaling

## Abstract

**Background:**

Patients affected by heat stroke (HS) develop myocardial injury at an early stage and exhibit a significantly higher risk of death than those without myocardial injury.

**Methods:**

We used WGCNA and myocardial tissue transcriptome sequencing to identify candidate DEGs associated with HS-induced myocardial injury. Immune infiltration and functional enrichment analyses were performed to investigate the correlation between candidate DEGs and immune cell populations and their biological functions. Protein–protein interaction (PPI) network analysis was used to identify hub genes. Clinical validation was performed through ELISA of blood samples from patients with HS, followed by construction of a hub gene-based prognostic nomogram. Additionally, the L1000FWD platform was used to screen potential small-molecule therapeutic drugs. Finally, we established HS mice models and cellular models to validate the therapeutic efficacy and underlying mechanisms of the selected compounds.

**Results:**

Thirteen candidate DEGs were identified in the HS myocardial tissues. Immune infiltration analysis showed significant positive correlations between these DEGs and macrophages, NK cells, and dendritic cells. Functional enrichment analysis indicated that the candidate DEGs were predominantly enriched in the MAPK signaling pathway. PPI network analysis identified JUN as a key hub gene in HS-induced myocardial injury. Clinical validation showed that c-Jun levels were significantly elevated in patients with than in those without HS myocardial injury (p < 0.001) with an area under the curve (AUC) of 0.781 that indicated diagnostic accuracy. A prognostic nomogram based on c-Jun achieved an AUC of 0.906 for predicting patient outcomes. Furthermore, the L1000FWD platform identified ZG-10 as a potential therapeutic drug. *In vivo* and *in vitro* experiments showed that ZG-10 improved cardiac function in HS mouse models, alleviated c-Jun-mediated inflammatory responses and apoptosis in myocardial tissues, and inhibited the JNK/p38 MAPK pathway to downregulate c-Jun expression.

**Conclusions:**

This study has systematically elucidated the central role of c-Jun in HS-induced myocardial injury. We have provided a novel biomarker for early diagnosis and prognostic evaluation of HS-induced myocardial injury. Additionally, we have identified ZG-10 as a potential therapeutic drug for HS-induced myocardial injury, which is a new treatment strategy for this condition.

## Introduction

Recently, global warming has led to a continuous increase in the frequency, intensity, and duration of extreme heat events during summer (1), which have resulted in a significant rise in heat-related diseases worldwide (2). Heat stroke (HS) is the most severe type of heat-related illness. It is a life-threatening condition caused by prolonged exposure to high-temperature environments and is characterized by central nervous system dysfunction, rapid increase in core body temperature (typically >40°C), and multi-organ dysfunction including cardiovascular, hepatic, and renal impairments (3). The case fatality rate remains alarmingly high and ranges from 26.5–63.2% despite modern medical interventions (4).

Heat exposure increases the risk of cardiovascular mortality (5). The heart is one of the primary organs initially damaged in HS (6), as evidenced by approximately 43.4–74.6% of patients with HS exhibiting cardiovascular dysfunction (7–10). Cardiovascular abnormalities often manifest early in HS and are characterized by heart failure, focal myocardial necrosis, and arrhythmias (6). A meta-analysis of patients with HS showed that pre-existing cardiovascular disease induced a significant 2.5-fold increase in risk of mortality (11). Moreover, survivors of HS may develop long-term cardiovascular sequelae (12). These findings highlight the profound impact of cardiac injury on the prognosis of patients with HS and the need for focused studies on therapeutic or preventive strategies for HS-induced myocardial injury. Currently, rapid cooling remains the only effective intervention for HS in clinical practice (4). However, no definitive treatment exists for HS-induced myocardial injury. Although aspirin alleviates heat-induced myocardial injury in avian cardiac tissues, it does not show any clinical benefits and may exacerbate liver dysfunction (13). HS is often accompanied by systemic inflammatory response during its progression (14) and shares pathophysiological mechanisms with sepsis (15). During HS progression, systemic inflammatory activation occurs through excessive release of pro-inflammatory cytokines such as interleukin (IL)-1β, IL-6, IL-2, and tumor necrosis factor-α (TNF-α) (16). These cytokines stimulate granulocyte migration and adhesion, which triggers an inflammatory cascade that leads to tissue damage (17). Simultaneously, injured myocardial tissue releases pro-inflammatory factors, which further amplify the systemic inflammatory response (16). Therefore, suppression of inflammatory response is a promising therapeutic approach for mitigating HS-induced myocardial injury.

The JUN gene is located on chromosome 1 and encodes the c-Jun transcription factor protein (18, 19). c-Jun is an oncogene and one of the most active transcriptional activators in the Jun family (20). c-Jun forms a dimeric complex with other proteins to constitute the activator protein-1 (AP-1) transcription factor, which responds to a variety of extracellular or intracellular stimuli by regulating gene expression, inflammatory responses, and cellular functions (21). c-Jun N-terminal kinase (JNK) is a member of the mitogen-activated protein kinase (MAPK) family. It is activated by oxidative stress, heat stress, and radiation (22). JNK promotes the phosphorylation of the transcription factor c-Jun, thereby inducing cell apoptosis or survival (23–25). In cardiomyocytes, JNK and its downstream target c-Jun are phosphorylated and activated. Subsequently, they participate in myocardial injury induced by ischemia-reperfusion, sepsis, and drug exposure (26–29). However, findings on the role of c-Jun in HS-induced myocardial injury are limited.

**Figure 1:**
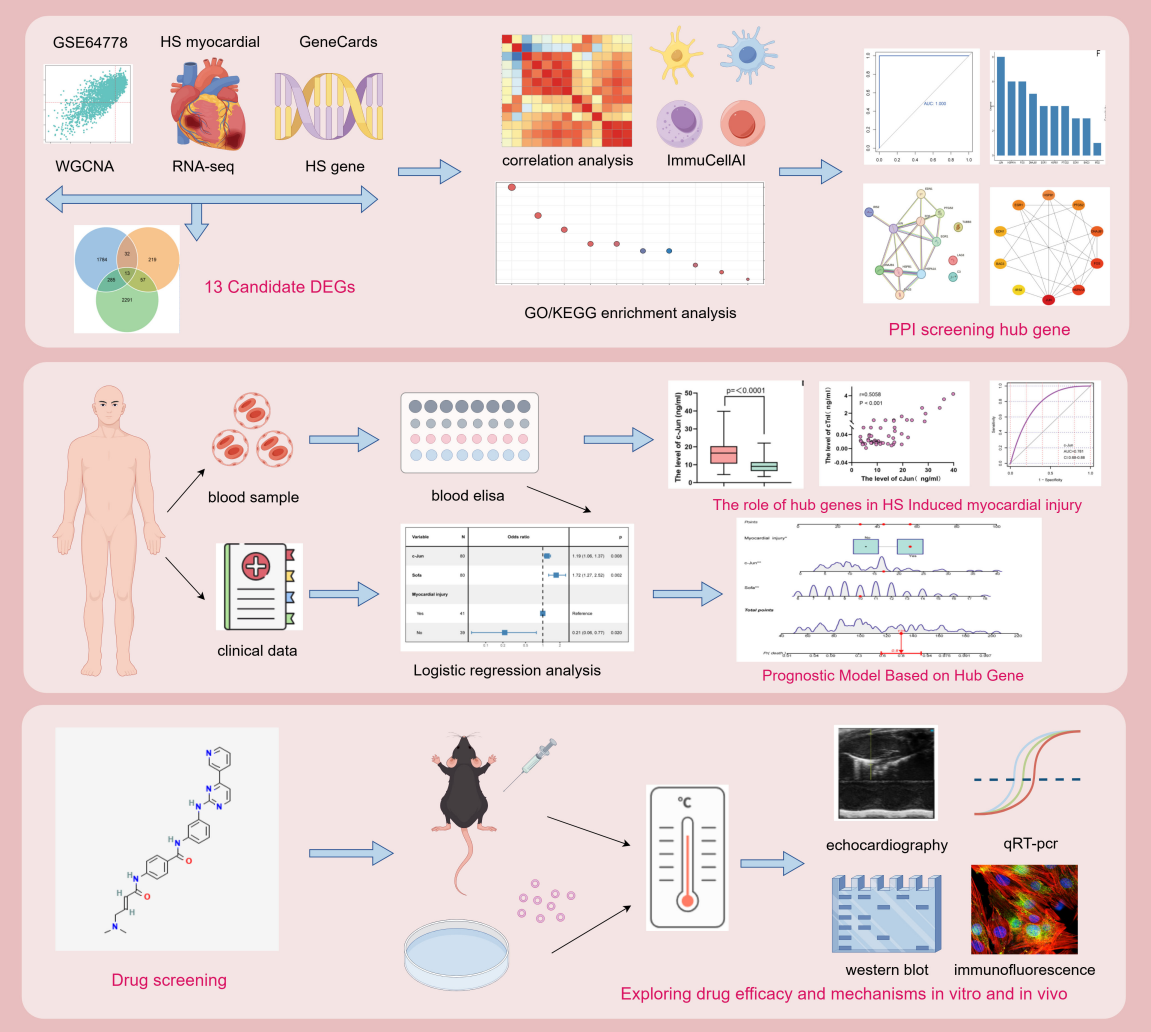
Study flowchart.

In this study, we performed bioinformatic analysis using public databases and sequencing data from HS myocardial tissues to identify key genes associated with HS-induced myocardial injury. Furthermore, we validated these findings in clinical samples, and c-Jun was identified as a diagnostic biomarker for HS-induced myocardial injury; additionally it showed predictive value for the prognosis of patients with HS. Finally, we used the identified key genes to screen for potential therapeutic drugs for HS-induced myocardial injury. Furthermore, we validated the efficacy of the identified drug by evaluating the therapeutic effects of ZG-10 both *in vivo* and *in vitro*. Additionally, we elucidated the potential underlying mechanisms. The study flowchart is presented in **Figure 1**.

## Materials and methods

### Acquisition of HS datasets from GEO database and preprocessing

We accessed the Gene Expression Omnibus (GEO) database and downloaded the HS-related transcriptomic dataset GSE64778. Specifically, this dataset includes whole-blood samples from 6 normal control and 16 HS rats induced with high temperature (39°C). The raw sequencing data of the GSE64778 dataset were obtained from the Sequence Read Archive (SRA). Next, fastp tool was used to perform quality control and preprocess the raw sequencing data to remove low-quality sequences and adapter contamination and trim the reads. Subsequently, the high-quality reads were aligned to the rat reference genome using Hisat2 software to determine gene expression levels. Next, the featureCounts tool was used to calculate the expression level of each gene in the samples. The results were used to generate an expression matrix.

### Weighted gene co-expression network analysis

WGCNA is a bioinformatics method for constructing gene co-expression networks based on gene expression data. We calculated the expression correlation between genes and assigning weights. This was used to cluster genes with similar expression patterns into modules. WGCNA is widely applied for biomarker identification and drug target discovery (30). In this study, we used the “WGCNA” R package to analyze the 22 rat whole-blood samples in the GSE64778 dataset. First, hierarchical clustering was performed to identify potential outliers. Next, the scale-free topology fit index was analyzed to determine the appropriate soft threshold β. Based on the optimal β value, the topological overlap matrix (TOM) was calculated, and a hierarchical clustering dendrogram was constructed. The branches of the hierarchical clustering tree were divided into co-expression modules, and each module contained at least 30 genes. To merge similar-colored modules, the mergeCutHeight parameter was set to 0.25. Finally, the correlation and significance of all genes within each module with respect to the phenotypic traits were calculated, and the most significantly correlated and relevant modules were selected as core modules.

### Transcriptomic analysis of HS-induced myocardial tissue

Total RNA was extracted from HS mice, and its concentration and purity were assessed using a Nanodrop 2000 spectrophotometer. RNA integrity was evaluated using agarose gel electrophoresis, and the RNA Quality Number (RQN) was determined using the Agilent 5300 platform. mRNA was isolated from total RNA and reverse-transcribed into cDNA. Subsequently, adapter sequences were ligated onto the cDNA, and the adapter-ligated products were purified and size-selected. The size-selected products were amplified via PCR, and the final library was purified. After library preparation, sequencing was performed on the NovaSeq X Plus platform. Raw data were subjected to quality control and aligned against the reference genome using fastp and Hisat2. The sequencing data has been uploaded to the GEO database (GSE303366). To identify differentially expressed genes (DEGs), the “DESeq2” R package was used to perform statistical analysis, for which the threshold was set at |log_2_FC| > 1 and p-value < 0.05. The “ggplot2” and “pheatmap” R packages were used to generate volcano plots and heatmaps, respectively. Additionally, we retrieved the genes associated with HS from the GeneCards database (https://www.genecards.org/). Specifically, genes with a GeneCards correlation score ≥ 3 were considered HS-related targets. The “ggvenn” R package was used to screen candidate DEGs for HS-induced myocardial injury. The “pheatmap” and “corrr” R packages were used to visualize the correlation heatmap and network of candidate DEGs, respectively.

### Immune infiltration analysis and correlation between candidate DEGs and immune cells

ImmuCellAI is a tool that is based on the single sample Gene Set Enrichment Analysis (ssGSEA) method and is used to evaluate the abundance of 24 immune cell-types from gene expression data (31). The “ImmuCellAI” R package was used to assess immune cell infiltration in the HS and control groups from the GSE64778 dataset. The “corrplot” and “ggplotify” R packages were used to determine and visualize the correlations between immune cells, whereas the “ggplot2” R package was used to generate heatmaps to illustrate the correlations between candidate DEGs and immune cells.

### Functional enrichment and protein–protein interaction network analysis of candidate DEGs

To gain a deeper understanding of the biological significance and potential molecular regulatory mechanisms of the candidate DEGs in HS-induced myocardial injury, we performed Gene Ontology (GO) and Kyoto Encyclopedia of Genes and Genomes (KEGG) pathway enrichment analyses. Additionally, the “enrichplot” R package was used to visualize the top 5 enriched GO terms and the top 10 enriched KEGG pathways. In the enrichment analysis, p-value < 0.05 was considered statistically significant.

The candidate DEGs were imported into the STRING database (https://cn.string-db.org/) to obtain protein–protein interaction (PPI) network information. Subsequently, the PPI data were imported into Cytoscape software for analysis and visualization. The MCC algorithm was used to identify the top 10 genes with the highest scores. The colors and sizes of the visualized nodes represent the strength of the interaction with other proteins. Additionally, the candidate DEGs were ranked based on their degree values.

### Sample and study respondents

This study was approved by the Ethics Committee of Deyang People’s Hospital (2024-04-003-K01). We adhered to the guidelines of the Declaration of Helsinki. All participants were recruited between April 1, 2024 and October 31, 2024 at Deyang People’s Hospital. All participants provided informed consent (Clinical trial number: not applicable). HS was diagnosed based on the expert consensus on the diagnosis and treatment of HS in China (32). The diagnostic criteria were as follows—Medical history: (1) Exposure to high temperature and high-humidity environments and (2) High intensity exercise; Clinical manifestations: (1) Central nervous system dysfunction (e.g., coma, convulsions, delirium, and abnormal behavior), (2) Core temperature > 40°C, (3) Multiple organ dysfunction (≥ 2), and (4) Severe coagulopathy or disseminated intravascular coagulation. HS diagnosis was considered when any one item from the medical history and any one item from the clinical manifestations lists were satisfied and the condition could not be explained with other reasons. Exclusion criteria included pregnant women; patients with a history of cardiovascular disease (such as coronary heart disease, cardiomyopathy, valvular heart disease, congenital heart disease, arrhythmia, and acute coronary syndrome) and immune-related diseases; and patients with incomplete clinical data. Myocardial injury was defined as serum cardiac troponin I (cTnI) levels > 99^th^ percentile reference upper limit (33). As these reference limits are assay-dependent, we specified that all measurements had to be performed using the ADVIA Centaur System (Siemens). In this study, the specific cutoff value was cTnI > 0.04 ng/ml (ADVIA Centaur Systems, Siemens, Germany).

### Enzyme-linked immunosorbent assay

All blood samples were collected on day 1 after the patients were diagnosed with HS. The collected blood samples were centrifuged at 3000 rpm and stored at −80°C until required for further analysis. Total c-Jun level in human serum sample was measured using the Total c-Jun ELISA Kit (23176C, Cell Signaling Technology, USA).

### Establishment and evaluation of prognostic model

Clinical data and serum c-Jun concentrations in patient samples were obtained to identify the prognostic factors. We determined the independent prognostic factors for patients with HS by performing a univariate logistic regression analysis to select variables with statistical significance. Variables with p < 0.05 were included in the multivariate logistic regression analysis to identify the independent prognostic factors. Receiver operating characteristic (ROC) curves were plotted for these independent prognostic factors, and the area under the curve (AUC) was determined to evaluate their specificity and sensitivity. Subsequently, a prognostic nomogram was developed using the “rms” R package based on the identified independent prognostic factors. ROC curves were plotted, and AUC values were calculated. Additionally, calibration curves were constructed, and decision curve analysis (DCA) was performed to assess the nomogram. The DCA curves are used to guide clinical decisions such as identifying high-risk patients who need to receive interventions and low-risk patients who need to avoid unnecessary treatments (to prevent overtreatment). Thus, this evaluation method indicates the clinical utility of the prognostic model. A perfect calibration curve should overlap with the 45° diagonal line. In fact, higher concordance between the calibration curve and the 45° diagonal line indicates more accurate predictive ability of the model.

### Identification of candidate drugs

The L1000 Fireworks Display (L1000FWD) provides an interactive visualization of gene expression profiles for > 16,000 drugs and small molecules (34). We used the L1000FWD tool to analyze the DEGs that exhibited either high or low expression levels in HS-induced myocardial injury. Through this analysis, we screened drugs that showed opposite correlations with the candidate DEGs.

### Construction of HS animal model

This study used 6–8-week-old C57BL/6N male mice (body weight: 20–25 g) provided by DOSSY Experimental Animal Co. Ltd. (Chengdu, China). All mice were acclimated for 1 week under standard housing conditions including 22–25°C temperature, 12-h light/dark cycle, and free access to food and water. The mice were divided into five groups: Control, HS, HS+DMSO, HS+ZG-10, and HS+ZG-10+ANI. The Control group was maintained in a normal environment (22–25°C and 60–65% humidity) with fasting and water deprivation. The HS group was exposed to high temperature intervention in a climate-controlled chamber. The temperature was set to 40 ± 0.5°C, and humidity was maintained at 60–65%. During this period, the mice were fasted and water-deprived. Additionally, rectal temperature was measured every 30 min using a rectal thermometer until the core body temperature reached 42°C. At this point the mice were immediately transferred to a recovery environment (22–25°C; 60–65% humidity). The HS+DMSO group mice were administered intraperitoneal injections of DMSO (concentration <1%) for 7 days prior to high-temperature exposure. The final dose was administered 1 h before administering the heat challenge. The remaining procedures were same protocol as those followed for the HS group. The HS+ZG-10 group mice were administered intraperitoneal injections of ZG-10 (T26349, Targetmol, USA) at a dose of 10 mg/kg for 7 days before high-temperature exposure. The final dose was administered 1 h before administering the heat challenge; the remaining procedures were the same as those followed for the HS group. The HS+ZG-10+ANI group mice were administered intraperitoneal injections of ZG-10 and ANI (S7409, Selleck, Shanghai, China) for 7 days before high-temperature exposure. The ZG-10 dose was the same as that used earlier, and the ANI dose was 50 mg/kg. The final dose was administered 1 h before administering the heat challenge, and the remaining procedures were the same protocol as those followed for the HS group. All animal experiments were conducted in accordance with the National Institutes of Health Guidelines on the Use of Laboratory Animals and were approved by the Animal Welfare Ethics Committee of Chongqing University Affiliated Central Hospital (2411005, Chongqing, China). Maximum effort was made to reduce the number of animals and their suffering.

### Echocardiographic assessment of cardiac function in mice

A depilatory cream was applied to the left precordial area of the mice to remove hair, followed by anesthesia with isoflurane. A sufficient amount of coupling agent was applied to the left precordial region, and a linear array transducer was used to locate the parasternal long-axis view of the left ventricle. The mouse heart was assessed using real-time echocardiogram. M-mode echocardiography was used to measure cardiac output (CO), left ventricular stroke volume (SV), left ventricular posterior wall thickness during systole (LVPWs), and left ventricular posterior wall thickness during diastole (LVPWd). Each group included five mice, and three consecutive cardiac cycles were measured per mouse.

### Cell culture and intervention

H9C2 rat cardiomyocytes were purchased from the Chinese Academy of Sciences Cell Bank. The cells were cultured in DMEM (SH30243.02, HyClone, USA) supplemented with 10% FBS (A5256701, Gibco, USA) and 1% penicillin–streptomycin (15140148, Thermo Scientific, USA). The H9C2 cells were maintained under standard conditions of 37°C and 5% CO_2_. According to the experimental design, the H9C2 cells were divided into five groups: Control, Heat Stress, Heat Stress+DMSO, Heat Stress+ZG-10 and Heat Stress+ZG-10+ANI. The Control group cells were cultured at 37°C. The HS group cells were exposed to high-temperature intervention at 43°C for 2 h, followed by recovery at 37°C for 3 h (35). The HS+DMSO group received DMSO (concentration <0.1%) prior to high-temperature intervention. The HS+ZG-10 group was treated with ZG-10, which was dissolved and diluted to the following concentrations: 5, 10, 20, 50, 100, and 500 μM. Then, ZG-10 was added to 96-well plates and incubated at 37°C or 43°C for 2 h to screen for the optimal concentration. The HS+ZG-10+ANI group was treated with ZG-10 (20μM) and ANI (1μM) prior to high-temperature intervention.

### Cell counting Kit-8 assay

The CCK-8 assay (C0038, Beyotime, Shanghai, China) was used to assess cardiomyocyte viability. Briefly, 5,000 cells were seeded onto 96-well plates according to the manufacturer’s instructions. H9C2 cells under different temperature conditions were treated with different concentrations of ZG-10. After intervention, CCK-8 reagent (10 μL) was added to each well, and the plates were incubated at 37°C for 2 h. Finally, the optical density (OD) at 450 nm was measured using a microplate reader.

### RNA extraction and analysis

Total RNA was extracted from tissues or serum using an RNA extraction kit (R0017S, Beyotime, Shanghai, China). Then, RNA concentration and purity were measured using a spectrophotometer with the A_260_/A_280_ ratio ranging from 1.8–2.0. The extracted total RNA was reverse transcribed into cDNA using a reverse transcription kit (RR047A, TaKaRa, Osaka, Japan). Quantitative RT-PCR was performed using the SYBR^®^ Premix Ex Taq™ kit (DRR041A, TaKaRa, Osaka, Japan). β-actin was used as the internal control for mRNA expression, and the relative gene expression levels were calculated using the 2^−ΔΔCt^ method. The mRNA primer sequences are listed in **Supplementary Table 1**.

### TUNEL fluorescent staining

The TUNEL cell apoptosis detection kit (C1088, Beyotime, Shanghai, China) was used according to the manufacturer’s instructions. H9C2 cells or mouse cardiac tissue sections were fixed with 4% paraformaldehyde, washed with PBS buffer, and permeabilized with Triton-X-100. After washing, the samples were equilibrated in the equilibration buffer for 10 min. Next, they were incubated at 37°C in the TUNEL reaction mixture for 60 min, followed by washing thrice in PBS. Finally, the samples were mounted with anti-fluorescence quenching mounting medium, and the results were observed and photographed under a fluorescence microscope.

### Immunofluorescence staining

Cells on coverslips or frozen heart sections were fixed with paraformaldehyde, washed thrice with PBS buffer, permeabilized with 0.5% Triton X-100 for 30 min, and blocked with 0.5% casein for 1 h at room temperature. Next, the cells were incubated overnight at 4°C with the primary antibodies against c-Jun, cTnT, IL-1β, and p-p38 MAPK. The next day, the appropriate fluorescent secondary antibodies were added and incubated at room temperature, followed by staining with DAPI. Finally, the sections were observed under a fluorescence microscope or confocal microscope.

### Western blotting

Total protein was extracted from the tissues or cells using RIPA lysis buffer (P0013K, Beyotime, Shanghai, China) containing protease inhibitors and phosphatase inhibitors (78440, Thermo Scientific, USA). BCA was quantified by performing a BCA protein assay (P0011, Beyotime, Shanghai, China). Identical quantities of protein samples were electrophoresed on a 10% SDS-PAGE gel and transferred onto PVDF membranes (IPVH00010, Millipore, USA), which were blocked using 5% BSA for 60 min at room temperature. Then, the blots were incubated overnight at 4°C with primary antibodies against Cleaved Caspase-3 (9579T, Cell Signaling Technology, USA), Caspase-3 (14220T, Cell Signaling Technology, USA), Bax (5023T, Cell Signaling Technology, USA), Bcl-2 (3498T, Cell Signaling Technology, USA), c-Jun (9165T, Cell Signaling Technology, USA), JNK (4668T, Cell Signaling Technology, USA), P-JNK (4671T, Cell Signaling Technology, USA), p38 MAPK (8690T, Cell Signaling Technology, USA), P-p38 MAPK (9211S, Cell Signaling Technology, USA), and β-actin (4967S, Cell Signaling Technology, USA). Next, the blots were probed with HRP-conjugated secondary anti-rabbit antibody (31460, Thermo Scientific, USA) or anti-mouse antibody (31430, Thermo Scientific, USA) at room temperature for 1 h. The bands were visualized using an enhanced chemiluminescent substrate (P0018AM, Beyotime, Shanghai, China) detection system (Image Lab, Bio-Rad). Quantification was performed using Image J Software, followed by statistical analysis.

### Statistical analysis

Statistical analysis was performed using GraphPad Prism (version 8.0) and R v4.3.2 software. First, the Shapiro–Wilk test and Levene’s test were performed to assess the normality and homogeneity of variance, respectively. If the data met the criteria for normal distribution and equal variance, two-group comparisons were performed using a t-test, and multiple-group comparisons were performed using ANOVA. For data that did not meet the assumptions of normality, non-parametric tests were applied. Spearman correlation analysis was used to assess associations between variables. Univariate and multivariate logistic regression analyses were performed to examine the relationships between independent variables and outcome variable. A p-value < 0.05 was considered statistically significant.

## Results

### Identification of HS-related genes via WGCNA

**Figure 2:**
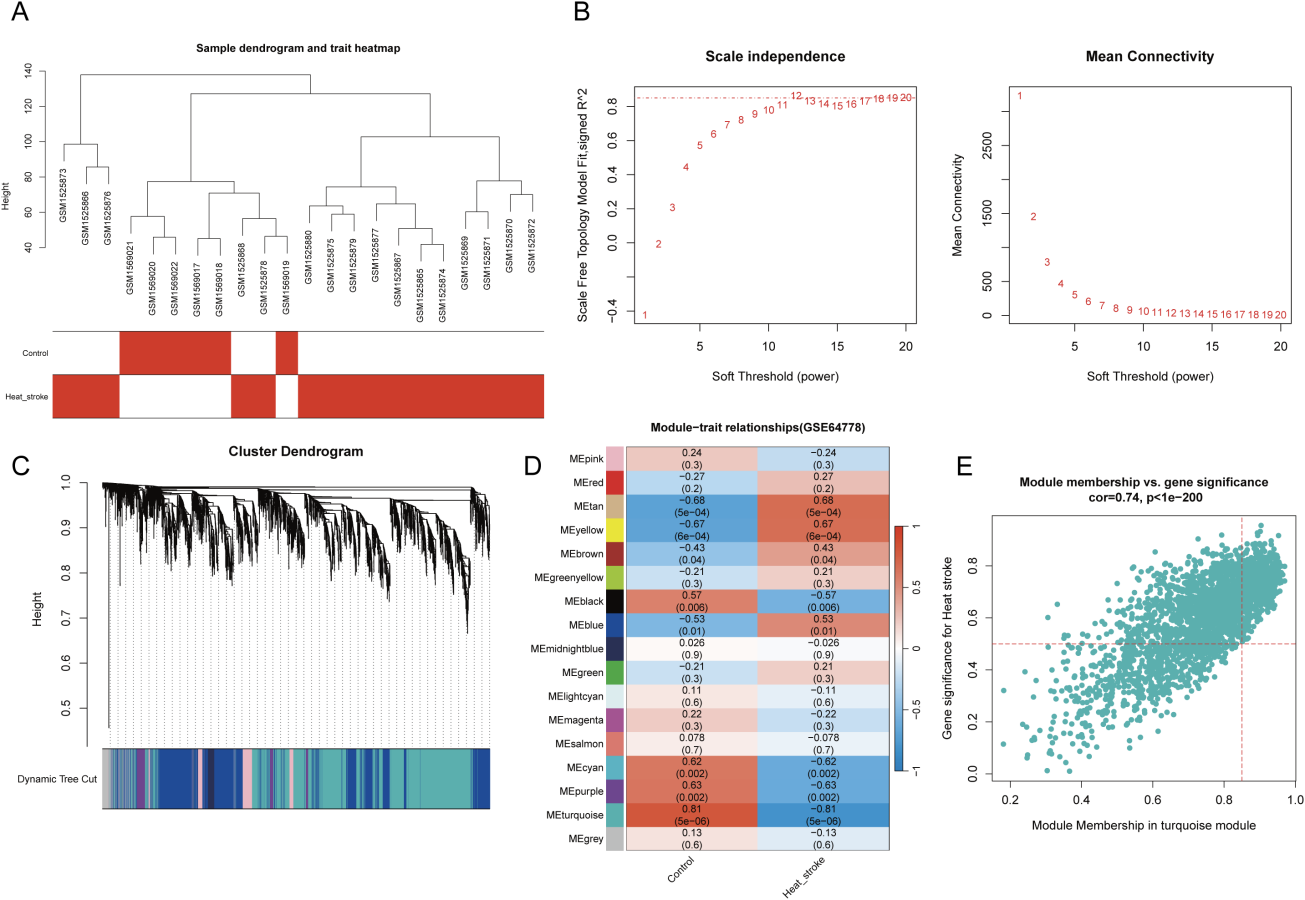
WGCNA identification of module genes associated with HS. **(A)** Clustering dendrogram of samples. **(B)** Scale independence and mean connectivity for various soft threshold powers β, the red line represents R2=0.85. **(C)** Gene clustering dendrogram. **(D)** Phenotype gene correlation heatmap, red represents positive correlation, blue represents negative correlation; the numbers in each cell represent correlation and significance. **(E)** Correlation analysis between turquoise module and phenotype.

First, a WGCNA was performed on the public dataset GSE64778 related to HS. In total, 22 samples were included in this analysis (HS group, n = 16; Control group, n = 6). Hierarchical clustering analysis of the samples showed no obvious outliers in the clustering dendrogram (**Figure 2A**). Subsequently, a soft threshold of β = 12 (scale-free R² = 0.85) was selected to construct the scale-free network (**Figure 2B**). Based on the optimal β value, a gene co-expression matrix was built, and module clustering was performed to generate a gene clustering dendrogram (**Figure 2C**). The relationship between trait data and gene modules was calculated. As a result, 17 co-expressed modules were identified (**Figure 2D**). Among these, the turquoise module (containing 2114 genes) showed the closest correlation with HS (**Figure 2E**).

### Identification of candidate DEGs associated with HS-induced myocardial injury

**Figure 3:**
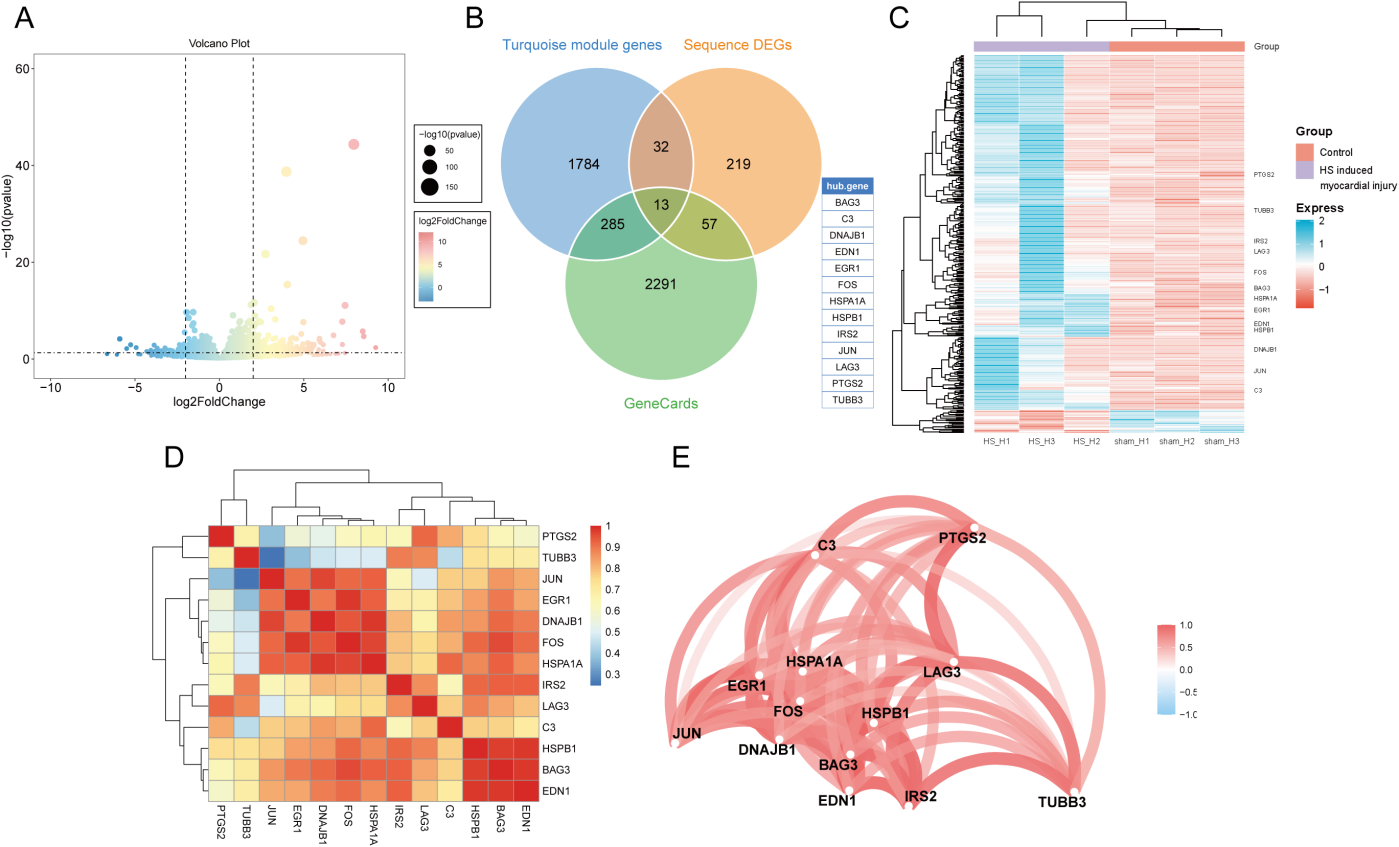
Identification of candidate DEGs associated with HS-induced myocardial injury. **(A)** Volcanic map of DEGs in HS myocardial tissue transcriptome sequencing. **(B)** The Venn diagram shows the intersection of the turquoise module genes, HS-related genes obtained from the GeneCards database, and DEGs from HS myocardial tissue sequencing. **(C)** Heatmap of DEGs in HS myocardial tissue transcriptome sequencing. **(D)** Correlation heatmap of candidate DEGs. **(E)** Correlation network diagram of candidate DEGs.

Next, we constructed an HS mouse model and performed transcriptome sequencing analysis on the myocardial tissues of the HS mice. Based on the predefined thresholds, 321 DEGs were identified in the myocardial tissues of the control and HS groups. Of these, 20 were downregulated and 301 were upregulated genes. The DEGs were visualized using a volcano plot (**Figure 3A**). The intersection of the turquoise module genes, HS-related genes obtained from the GeneCards database, and DEGs from HS myocardial tissue sequencing helped identify 13 candidate DEGs (**Figure 3B**). These were BAG3, DNAJB1, EDN1, EGR1, FOS, HSPA1A, HSPB1, IRS2, JUN, LAG3, PTGS2, TUBB3, and C3. A heatmap was constructed, and it illustrated the expression levels of these candidate DEGs in the myocardial tissues from the control and HS groups; additionally, all candidate DEGs showed high expression in the HS myocardial tissues (**Figure 3C**). Furthermore, the correlation heatmap and network diagram showed that the expression of all candidate DEGs was positively correlated (**Figures 3D, E**).

### Correlation of immune infiltration with candidate DEGs

**Figure 4:**
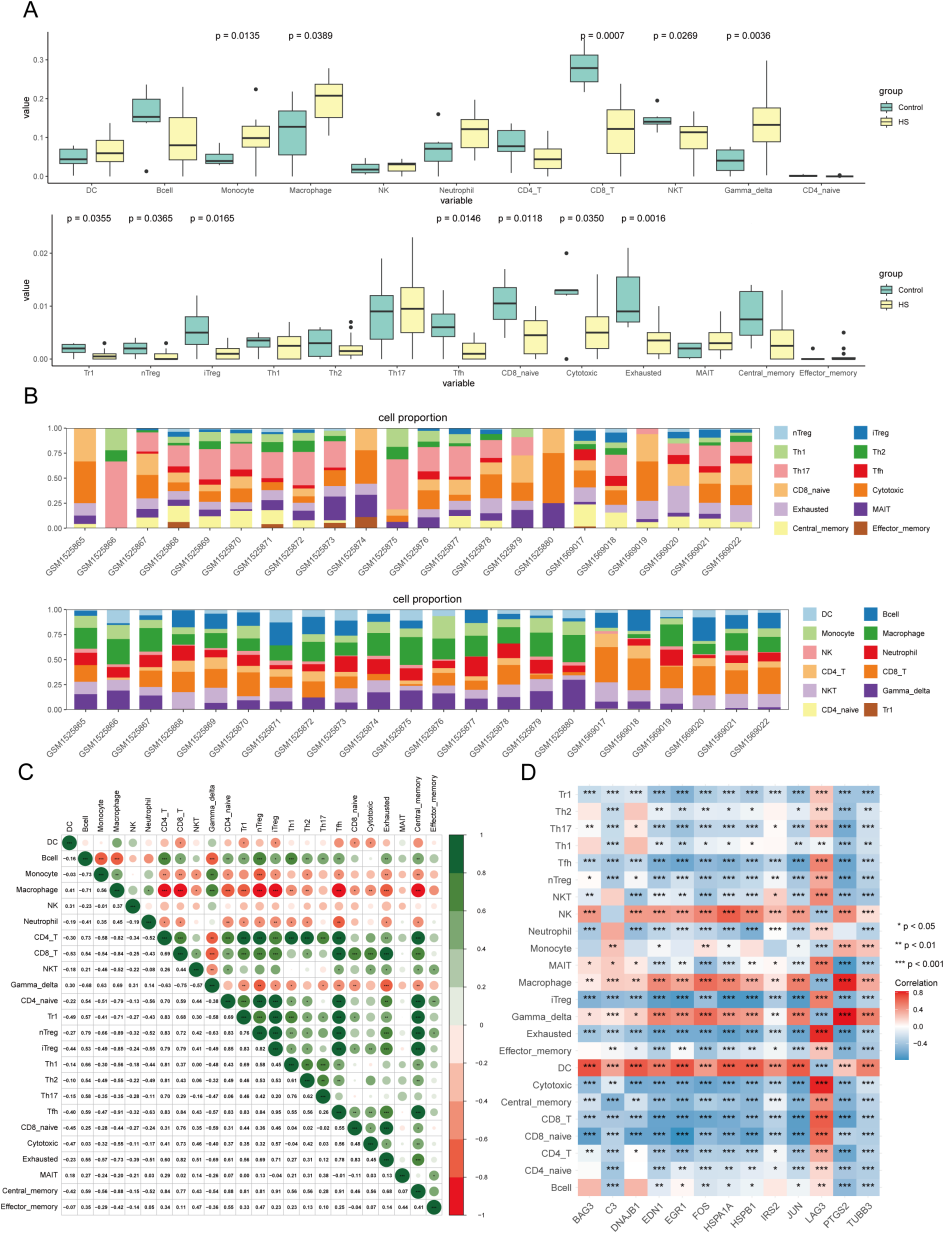
Immune infiltration analysis of candidate DEGs. **(A)** The box plot illustrates the levels of immune cell infiltration and their differential patterns between HS and normal samples. **(B)** Cluster heatmap of 24 immune cell proportions in GSE64778 dataset. **(C)** Matrix diagram of 24 immune cell correlations. green denote positive associations, red indicate inverse relationships, and chromatic intensity scales with correlation magnitude. **(D)** Heatmap of the correlation between candidate DEGs and immune cells, asterisk indicating the significance of the correlation.

Heat stress activates the immune system, which leads to significant changes in the quantity and function of immune cells to maintain homeostasis (36–38). Therefore, we evaluated the correlation between immune cell infiltration and candidate DEGs. We found that 12 of the 24 immune cell-types exhibited significant differences between the control and HS groups (**Figures 4A, B**). Additionally, significant correlations were observed among the 24 immune cell types (**Figure 4C**). Specifically, the HS group was enriched in monocytes (fold change: 2.49), macrophages (fold change: 1.62), and gamma delta T cells (old change: 3.27), whereas the control group showed higher levels of CD8^+^ T cells (fold change: 2.28), NKT cells (fold change: 1.24), and Tr1 cells (fold change: 4). To elucidate the relationship between the candidate DEGs and individual immune cells, we constructed a correlation heatmap, which showed that that candidate DEGs positively correlated with macrophages, NK cells, and DCs but negatively correlated with Tr1 cells, CD8^+^ T cells, exhausted T cells, and cytotoxic T cells (**Figure 4D**).

### Functional enrichment analysis and identification of hub gene

**Figure 5:**
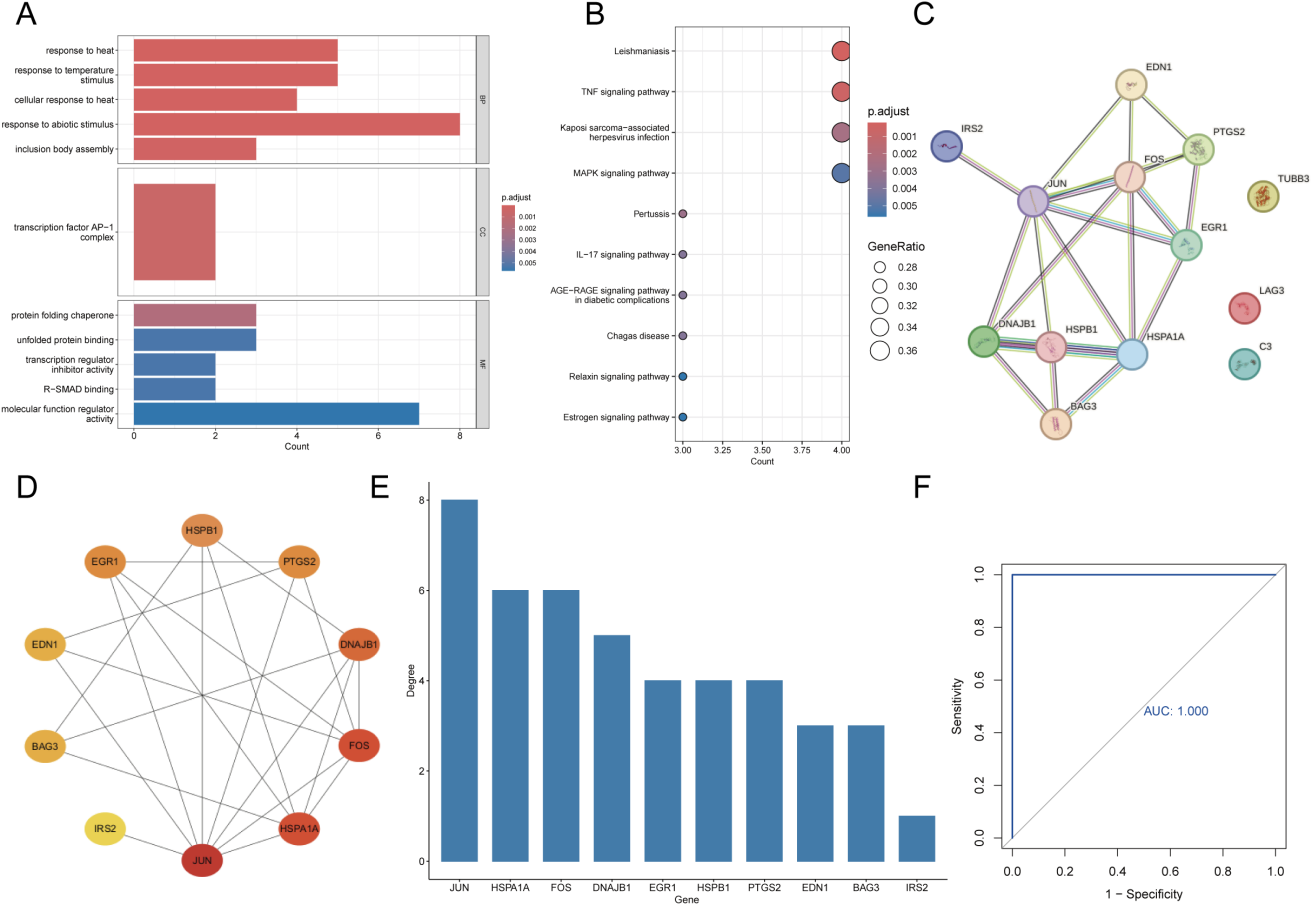
Functional enrichment analysis and identification of hub gene. **(A)** The top 5 GO enrichment analyses of biological processes, cellular component and molecular function. **(B)** The top 10 pathways for KEGG enrichment analysis. **(C)** Protein-protein interaction network diagram from the STRING online database. **(D)** The PPI network of candidate DEGs was constructed using Cytoscape software, with the top 10 genes prioritized by the MCC algorithm. **(E)** bar graph of the top 10 gene sorted by degree. **(F)** ROC curves for analyzing the accuracy of JUN in predicting the occurrence of HS.

We performed GO and KEGG enrichment analyses to identify the signaling pathways and biological functions associated with the candidate DEGs. GO enrichment analysis showed that the biological processes were primarily enriched in response to heat or temperature stimulus and response to abiotic stimulus. The analysis of cellular components showed a significant focus on transcription factor AP-1 complex. Additionally, terms such as protein folding chaperone, unfolded protein binding, and transcription regulator inhibitor activity were significantly enriched in the functional analysis (**Figure 5A**). KEGG analysis indicated that the candidate DEGs were predominantly enriched in pathways such as Leishmaniasis, TNF signaling pathway, Kaposi sarcoma-associated herpesvirus infection, and MAPK signaling pathway (**Figure 5B**). We identified the hub gene associated with HS-induced myocardial injury by importing the candidate DEGs into the STRING database and performing PPI analysis to obtain the protein interaction network information (**Figure 5C**). Next, the protein interaction data were imported into Cytoscape software, and the top 10 genes were identified using the MCC algorithm to construct the PPI network (**Figure 5D**). Based on the degree values, the candidate DEGs were ranked as follows: JUN, HSPA1A, FOS, DNAJB1, EGR1, HSPB1, PTGS2, EDN1, BAG3, and IRS2. Among these, JUN exhibited the highest degree value and was identified as the hub gene. Furthermore, JUN exhibited an AUC of 1 in the ROC curve for diagnosing HS, which indicates its exceptional accuracy in predicting HS occurrence (**Figures 5E, F**).

### Expression of hub gene and construction of prognostic models

**Figure 6:**
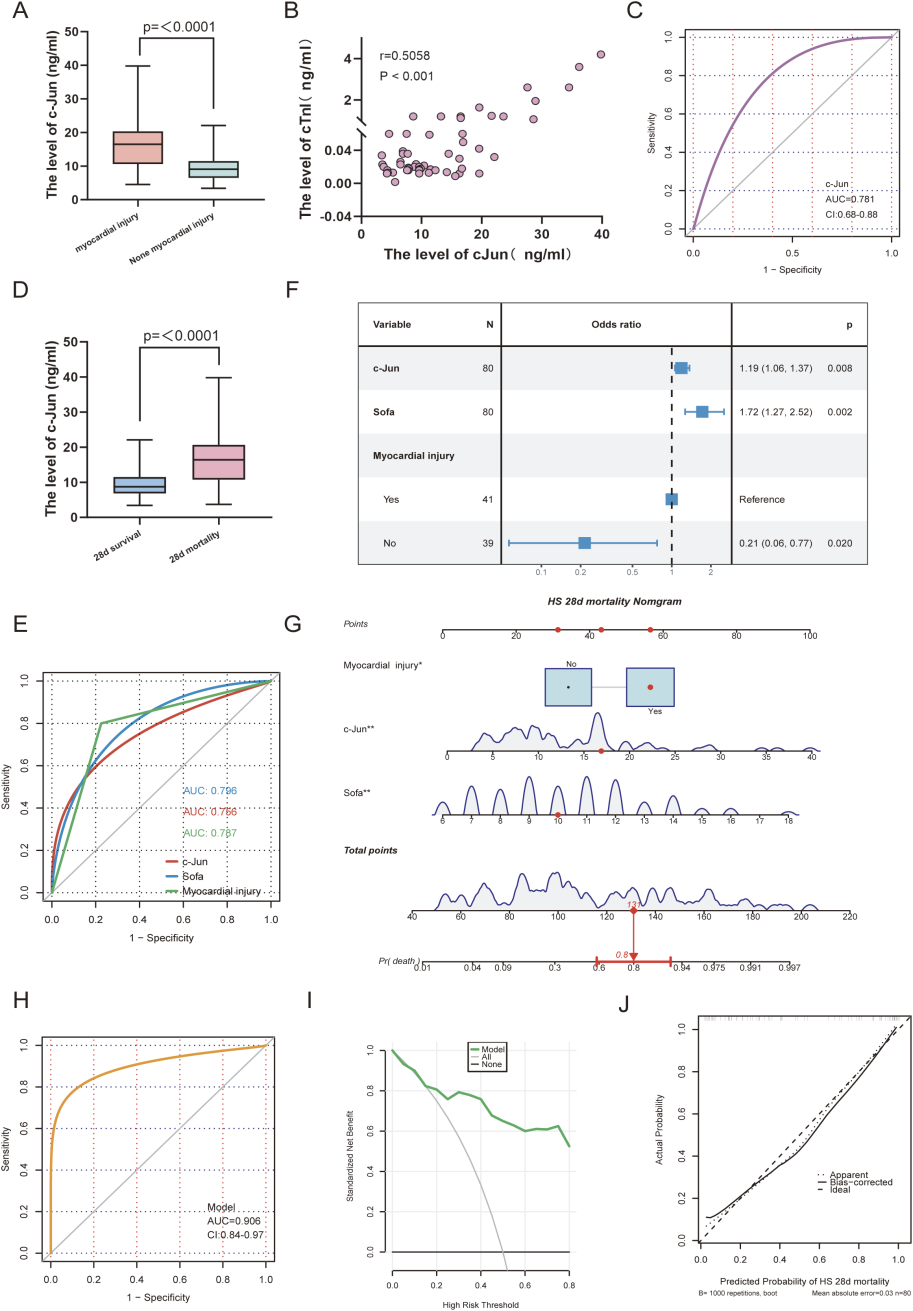
Expression of hub gene and construction of prognostic models. **(A)** The box plot shows the levels of c-jun in HS myocardial injury and non-myocardial injury. **(B)** Scatter plot illustrating the correlation between c-Jun and cTnI expression levels. **(C)** ROC curve analysis evaluating the diagnostic efficacy of c-Jun for myocardial injury in HS. **(D)** The box plot shows the levels of c-jun in HS 28d mortality and survivors. **(E)** ROC curve analysis evaluating the prediction efficacy of c-Jun, myocardial injury and sofa score in HS 28d mortality. **(F)** Forest plot of multivariate logistic regression analysis. **(G)** The nomogram for HS 28d mortality. **(H)** ROC curve analysis evaluating the prediction efficacy of nomogram in HS 28d mortality. **(I)** DCA curves are used to evaluation method for the clinical utility of the prognostic model. **(J)** Calibration curve are used to evaluation the predictive ability of the prognostic model. The closer the calibration curve aligns with the 45 diagonal line, the stronger the predictive performance.

In total, 80 patients with HS were enrolled in this study, including 41 patients with myocardial injury and 39 patients without myocardial injury. Initially, we compared the baseline characteristics of the patients with HS between those with and without myocardial injury (**Table 1**). The results showed that the patients with myocardial injury exhibited significantly higher levels of c-Jun and cardiac troponin I (cTnI) compared with those without myocardial injury (p < 0.001). Additionally, the Sequential Organ Failure Assessment (SOFA) score, which is used to evaluate the severity of multiple organ dysfunction syndrome (MODS) in critically ill patients, was significantly high in the myocardial injury group (p = 0.001). Notably, the 28-day mortality rate reached 78% in the myocardial injury group, whereas it was 20.5% in the non-myocardial injury group (p < 0.001). Box plots showed that c-Jun levels were significantly elevated in patients with HS presenting myocardial injury compared with that of those without (p < 0.001); furthermore, a positive correlation was observed between c-Jun and cTnI levels (r = 0.5058, p < 0.001), which indicates a strong association between c-Jun and severity of myocardial injury (**Figures 6A–B**). To validate the diagnostic potential of c-Jun in distinguishing HS-related myocardial injury, the AUC of the ROC curve was calculated and found to be 0.781, which suggests that c-Jun is a promising biomarker for myocardial injury in HS (**Figure 6C**). Furthermore, serum c-Jun levels were significantly higher in patients who died within 28 days compared with that of the survivors (p < 0.001) (**Figure 6D**). Next, we identified the prognostic factors for 28-day mortality in patients with HS by performing univariate logistic regression analysis by integrating clinical data, laboratory findings, and SOFA scores. The results indicated that the presence of myocardial injury, c-Jun levels, and SOFA scores were statistically significant predictors (**Supplementary Table 2**). Furthermore, we evaluated the predictive performance of these factors using ROC analysis. The AUC values for myocardial injury status, c-Jun levels, and SOFA scores were 0.787, 0.756, and 0.796, respectively, which indicates their robust predictive capabilities (**Figure 6E**). Additionally, a multivariate logistic regression analysis was performed to account for confounding variables. Forest plots confirmed that the presence of myocardial injury (p = 0.020), c-Jun levels (p = 0.008), and SOFA scores (p = 0.002) remained independent prognostic factors (**Figure 6F**). Based on the multivariate logistic regression results, we developed a nomogram to predict 28-day mortality in patients with HS. As indicated by the red markers in the nomogram, a patient with HS who has myocardial injury, a serum c-Jun level of 16.9 ng/ml, and a SOFA score of 10 would achieve a total score of 131, which corresponds to an 80% 28-day mortality risk (**Figure 6G**). The nomogram demonstrated excellent predictive accuracy with an AUC of 0.906 (95% CI: 0.84–0.97) (**Figure 6H**). To assess the clinical utility of the model, we generated decision curve analysis (DCA) and calibration curves. DCA results showed that the net benefit of the model consistently exceeded that of treating all patients (“All”) or none (“None”), which highlights its strong clinical applicability (**Figure 6I**). Calibration curves further confirmed the reliability of the model, as evidenced by the close alignment of the predicted probabilities with the observed outcomes along the 45° diagonal line (**Figure 6J**).

**Table 1:** Baseline characteristics of heat stroke patients.

Characteristic	HS without myocardial injury (N=39 )	HS induced myocardial injury (N=41)	p-value
C-Jun (ng/ml), Median (Q1, Q3)	9.06 [6.70-11.5]	16.5 [10.6-19.6]	<0.001
cTnl (ng/ml), Median (Q1, Q3)	0.02 [0.01-0.02]	0.26 [0.09-1.20]	<0.001
Sex, n (%)			0.273
Male	20 (51.3%)	27 (65.9%)	
Female	19 (48.7%)	14 (34.1%)	
Age, Median (Q1, Q3)	70.0 [58.5-79.0]	66.0 [52.0-78.0]	0.419
WBC (K/uL), Mean ± SD	15.3 (±6.01)	16.7 (±7.29)	0.343
PLT (K/uL), Median (Q1, Q3)	115 [58.5-156]	95.0 [59.0-130]	0.381
MONO (K/uL), Median (Q1, Q3)	0.92 [0.33-1.56]	0.92 [0.48-1.65]	0.59
NEUT (K/uL), Mean ± SD	13.4 (±5.51)	14.0 (±6.80)	0.656
Sofa, Mean ± SD	9.00 [7.00-11.0]	11.0 [9.00-13.0]	0.001
28-day mortality, n (%)	8 (20.5%)	32 (78.0%)	<0.001

WBC, white blood cell; PLT, platelet; MONO, monocyte; NEUT, Neutrophils.

### HS activates c-Jun-mediated inflammatory environment and apoptosis in myocardial tissue

**Figure 7:**
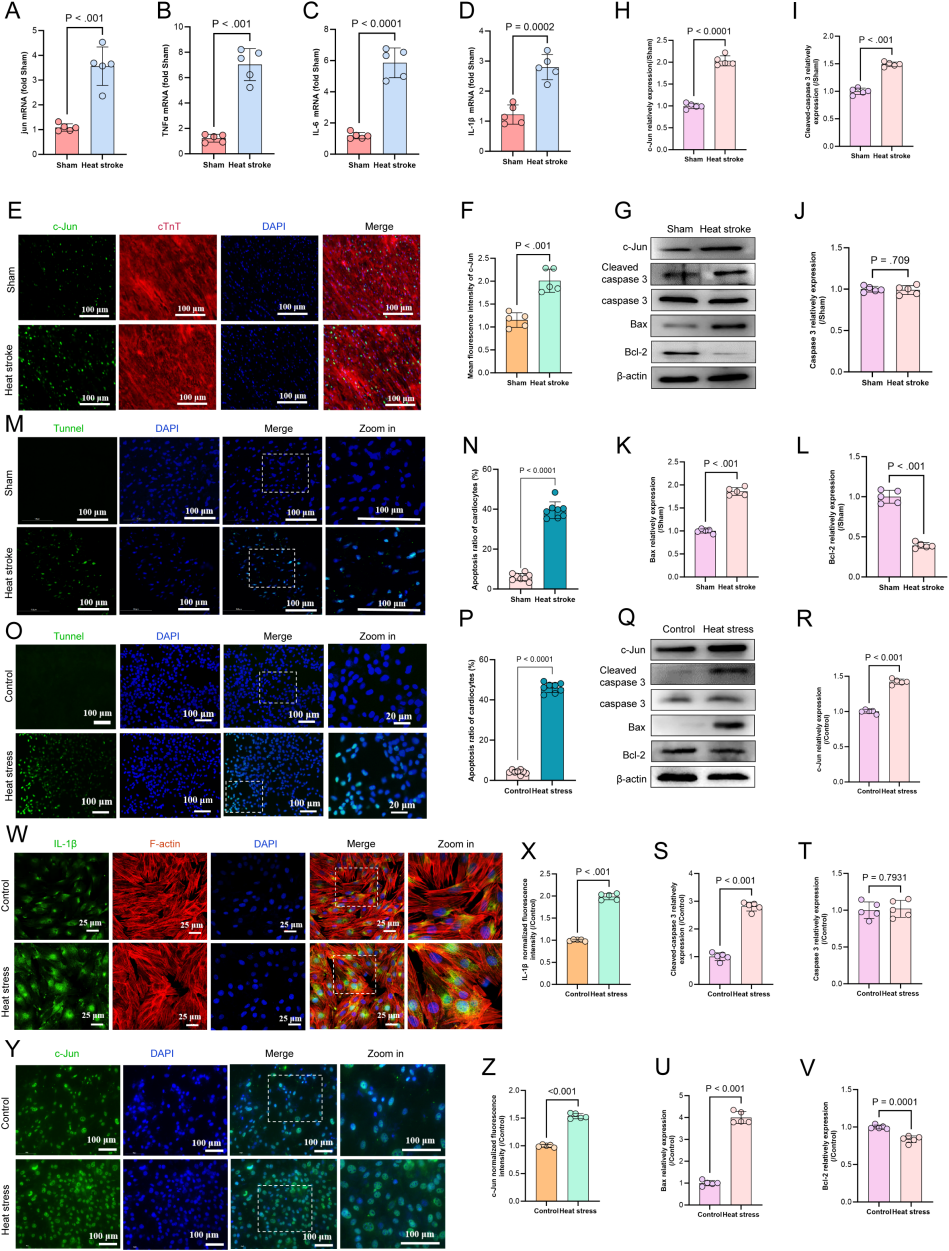
HS triggers c-Jun-mediated myocardial inflammation & apoptosis. **(A–D)** The mRNA levels of JUN, TNF-α, IL-6and IL-1β in myocardial tissue were determined using qRT-PCR, n = 5. **(E, F)** The fluorescence images and relative fluorescence intensity of c-Jun in myocardial tissue, scale bar = 100 μm, n = 5. **(G–L)** Representative western blot bands of c-Jun, cleaved-caspase 3, caspase 3, Bax and Bcl2 in myocardial tissue. Quantitative analysis of protein expression levels (normalized to β-actin), n = 5. **(M, N)** TUNEL staining of cardiomyocyte apoptosis in mice myocardial tissues across each groups. Quantitative analysis of apoptotic cells, scale bar = 100 μm, n = 8. **(O, P)** TUNEL staining of apoptosis in H9C2 cardiomyocytes across each groups. Quantitative analysis of apoptotic cells, scale bar = 100 μm, n = 8. **(Q–V)** Representative western blot bands of c-Jun, cleaved-caspase 3, caspase 3, Bax and Bcl2 in H9C2 cardiomyocytes. Quantitative analysis of protein expression levels (normalized to β-actin), n = 5. **(W–Z)** The fluorescence images and relative fluorescence intensity of IL-1β and c-Jun in H9C2 cardiomyocytes, scale bar = 25 μm/100 μm, n = 5.

The JUN gene encodes the c-Jun transcription factor. Upon activation, the transcription factor induces the expression of pro-inflammatory ligands such as FasL and TNF. This triggers the activation of caspase-3 and initiates apoptosis via the death receptor pathway. Additionally, c-Jun upregulates BH3-only proteins, activates pro-apoptotic proteins like Bax, and mediates mitochondrial pathway apoptosis. To investigate the changes in JUN expression and its role in HS-induced myocardial injury, we assessed relevant markers using qRT-PCR, western blotting, and immunofluorescence. The results showed that the mRNA levels of JUN, TNF-α, IL-6, and IL-1β were significantly elevated in the HS group compared with those in the Sham group (p < 0.01) (**Figures 7A–D**). Immunofluorescence analysis showed a pronounced increase in c-Jun fluorescence intensity in myocardial tissue (p < 0.01) (**Figures 7E, F**). Western blotting analysis confirmed the upregulation of c-Jun expression in HS-induced myocardial injury accompanied by significant increase in the expression of pro-apoptotic markers such as cleaved caspase-3 and Bax, whereas the expression of the anti-apoptotic protein BCL2 was downregulated (p < 0.01) (**Figures 7G–L**). TUNEL staining further validated the occurrence of apoptosis in HS-induced myocardial injury (p < 0.0001) (**Figures 7M–N**). These findings suggest that c-Jun is activated in HS-induced myocardial injury and mediates both inflammatory responses and apoptosis. To further validate this hypothesis, we subjected H9C2 cardiomyocytes to heat stress at 43°C, followed by recovery at 37°C for 3 h. Similar to that observed in the myocardial tissue, TUNEL staining confirmed that heat stress induced apoptosis in H9C2 cells (p < 0.0001) (**Figures 7O–P**). Moreover, heat-stressed H9C2 cells exhibited increased c-Jun expression compared with that in the control group accompanied by elevated levels of the pro-apoptotic markers cleaved-caspase 3 and Bax; additionally, the anti-apoptotic protein BCL2 was downregulated (p < 0.01), whereas inflammatory markers such as IL-1β were upregulated (p < 0.01) (**Figures 7Q–Z**). In summary, c-Jun is significantly upregulated in HS-induced myocardial injury along with the activation of apoptotic signaling pathways and presence of an inflammatory microenvironment.

### L1000 FWD screening for small-molecule drug

We used the L1000 FWD online platform to screen for drug candidates to target the DEGs associated with HS-induced myocardial injury. As all candidate DEGs were upregulated in HS-induced myocardial injury, we identified small molecules with opposite correlation profiles. The top 10 candidate drug are listed in **Table 2**. Wortmannin is a steroid metabolite derived from Penicillium funiculosum. It influences cell cycle progression and apoptosis by inhibiting the PI3K-AKT signaling pathway (39). Mitoxantrone, an anthracycline-like drug, is widely used in the treatment of various cancers. It functions by inhibiting DNA topoisomerase II (40). Tosedostat is an oral aminopeptidase inhibitor. It is primarily used in the treatment of acute myeloid leukemia (41). ZG-10 is a covalent inhibitor of c-Jun N-terminal kinase (JNK). ZG-10 irreversibly binds to its target protein via covalent bonding and induces conformational changes, which inhibit JNK activity (42, 43). After integrating drug ranking, potential mechanism of action and accessibility, ZG-10 was selected as the potential therapeutic drug for heat stroke-induced myocardial injury.

**Table 2:** The top ten candidate drugs of opposite relevance in the L1000FWD.

Drug	Similarity score	p-value	q-value	Z-score	Combined score
wortmannin	-0.7000	6.91e-11	2.14e-08	1.82	-18.53
SB-218078	-0.7000	8.78e-11	2.42e-08	1.81	-18.17
tyrphostin-AG-1478	-0.6000	5.55e-09	5.62e-07	1.81	-14.98
ZG-10	-0.6000	6.09e-09	5.63e-07	1.73	-14.21
GSK-1070916	-0.6000	6.26e-09	5.63e-07	1.73	-14.20
PP-110	-0.6000	4.78e-09	5.62e-07	1.84	-15.33
mitoxantrone	-0.6000	3.26e-09	5.62e-07	1.87	-15.90
staurosporine	-0.6000	7.59e-09	6.10e-07	1.66	-13.48
BRD-K05649647	-0.6000	4.53e-09	5.62e-07	1.82	-15.16
tosedostat	-0.6000	5.86e-09	5.62e-07	1.81	-14.92

### ZG-10 ameliorates HS-induced myocardial injury

**Figure 8:**
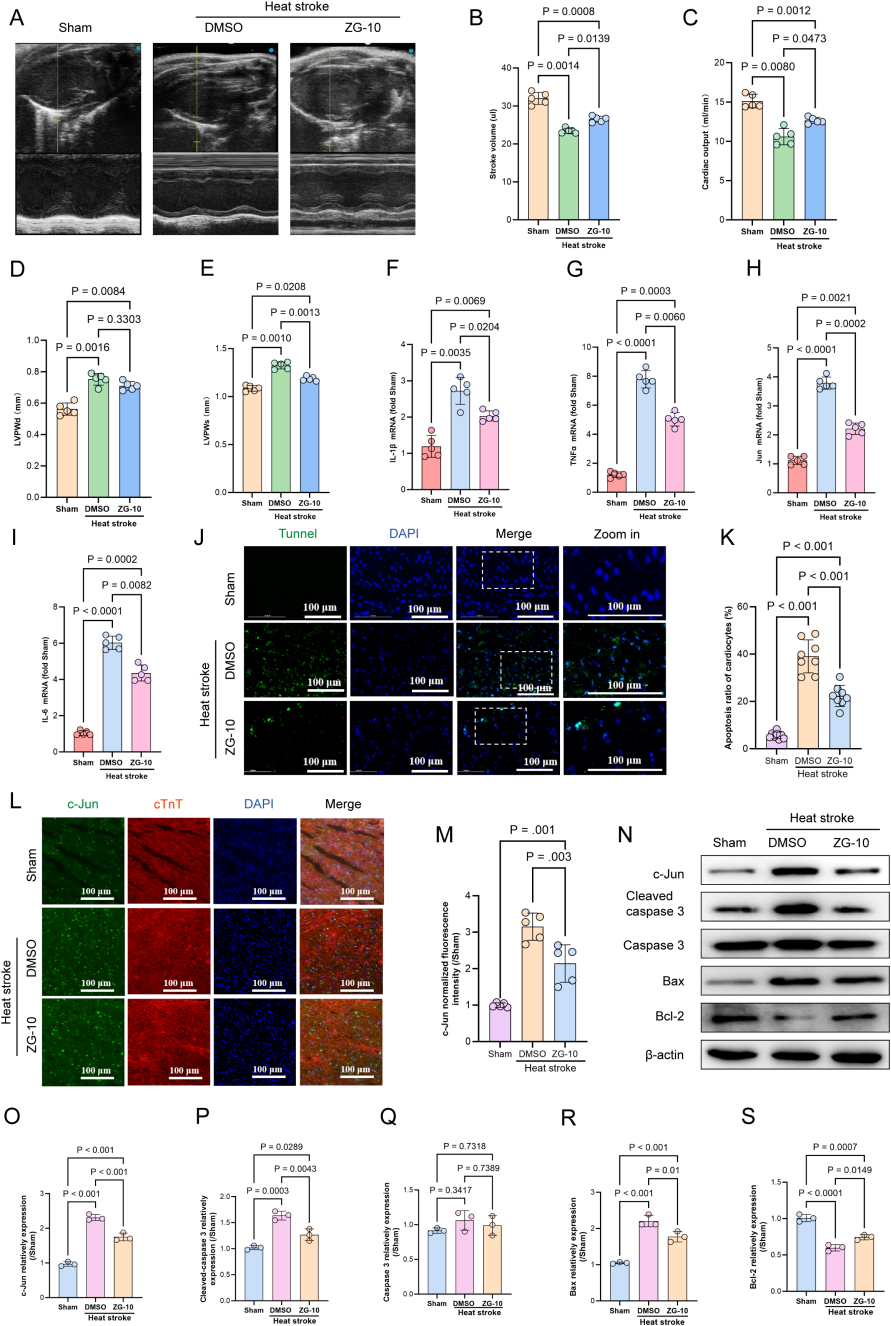
ZG-10 ameliorates HS-induced myocardial injury. **(A)** Echocardiographic assessment across experimental mice cohorts. **(B–E)** Statistical analysis of murine cardiac functional parameters: stroke volume (SV), cardiac output (CO), left ventricular posterior wall thickness in diastole (LVPWd) and systole (LVPWs), n=5. **(F–I)** The mRNA levels of JUN, TNF-α, IL-6and IL-1β in myocardial tissue were determined using qRT-PCR, n = 5. **(J, K)** TUNEL staining of cardiomyocyte apoptosis in mice myocardial tissues across each groups. Quantitative analysis of apoptotic cells, scale bar = 100 μm, n = 8. **(L, M)** The fluorescence images and relative fluorescence intensity of c-Jun in myocardial tissue, scale bar = 100 μm, n = 5. **(N–S)** Representative western blot bands of c-Jun, cleaved-caspase 3, caspase 3, Bax and Bcl2 in myocardial tissue. Quantitative analysis of protein expression levels (normalized to β-actin), n = 5.

JNK is a key regulator of apoptosis. Upon activation, JNK translocates from the cytoplasm to the nucleus and phosphorylates and activates the transcription factors such as c-Jun, c-Fos, and Elk-1, which results in the modulation of the expression of downstream apoptotic genes (44–46). Based on these mechanisms, we hypothesize that ZG-10 alleviates HS-induced myocardial injury by suppressing c-Jun expression. To validate this hypothesis, we administered ZG-10 (10 mg/kg) via intraperitoneal injection to mice 7 days before exposure to heat stress. First, we performed echocardiographic analysis. The results showed that the HS+DMSO group mice exhibited significant decrease in SV and CO (p < 0.05) compared with those of the Sham group mice along with substantial increase in LVPWd and LVPWs (p < 0.05). ZG-10 significantly improved cardiac function under heat stress, as evidenced by the significant increase in SV and CO (p < 0.05), substantial decrease in LVPWs (p < 0.05), and decreasing trend for LVPWd, although the difference was not statistically significant (p = 0.3303) (**Figures 8A–E**). qRT-PCR results showed that ZG-10 significantly downregulated JUN mRNA expression and suppressed the levels of the pro-inflammatory cytokines TNFα, IL-6, and IL-1β (**Figures 8F–I**). Furthermore, TUNEL staining confirmed that ZG-10 alleviated HS-induced myocardial apoptosis (**Figures 8J–K**). As c-Jun expression is significant in HS-induced myocardial injury and a potential predictive biomarker for myocardial damage severity and 28-day mortality in patients with HS, we performed immunofluorescence and western blotting analyses to assess c-Jun expression. We found that ZG-10 significantly inhibited c-Jun expression in HS-induced myocardial tissue (p < 0.05) (**Figures 8L–O**). Western blotting results confirmed that ZG-10 reduced the expression of pro-apoptosis proteins such as cleaved-caspase 3 and Bax, whereas it upregulated the anti-apoptotic protein BCL2 compared with that in the DMSO control group (p < 0.05) (**Figures 8P–S**). Collectively, these findings provide clear evidence that ZG-10 mitigates HS-induced myocardial injury by suppressing c-Jun-mediated apoptosis and inflammatory responses.

### ZG-10 ameliorates heat stress-induced cardiomyocyte injury

**Figure 9:**
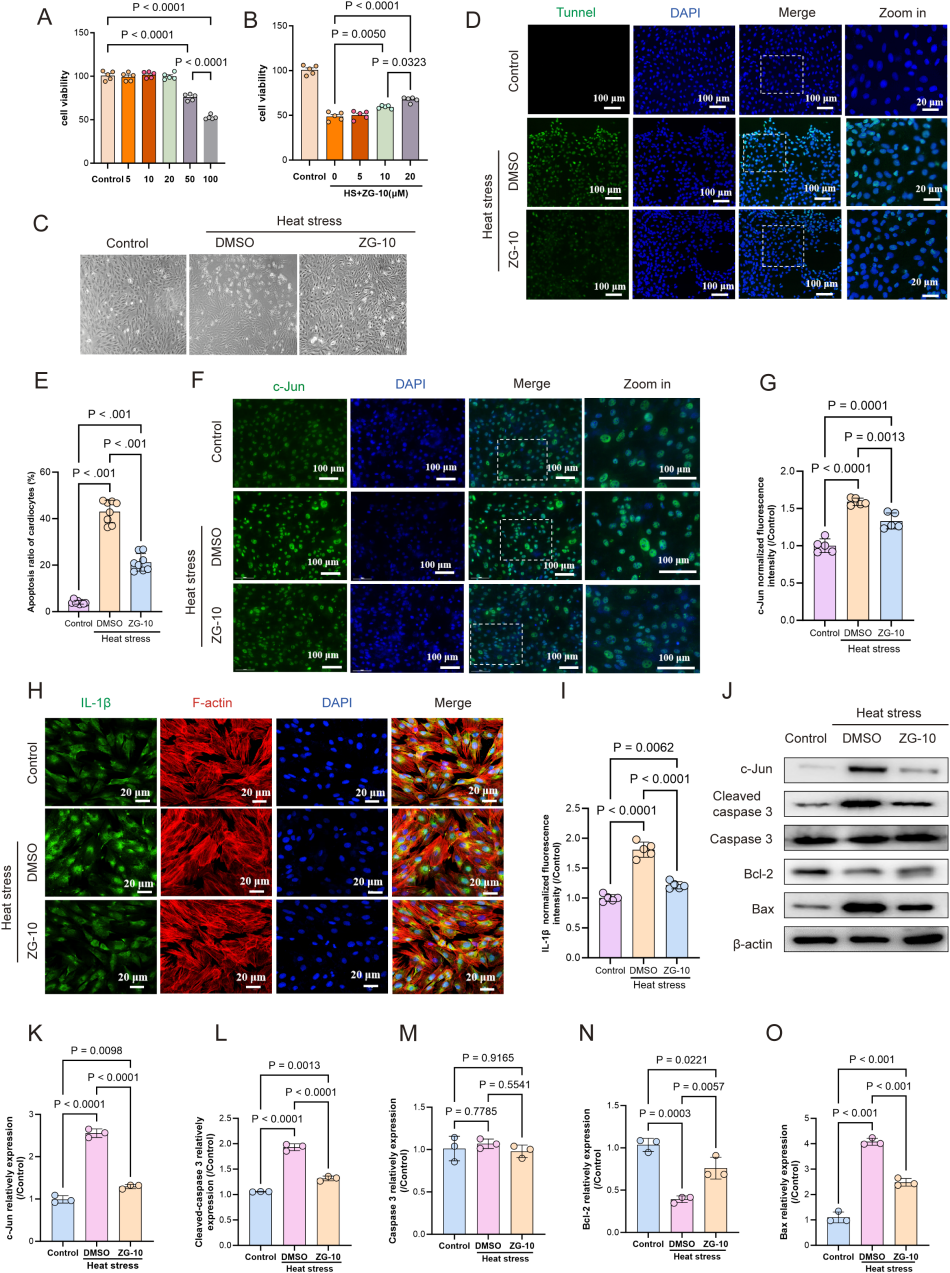
ZG-10 ameliorates heat stress-induced cardiomyocyte injury. **(A)** Concentration-dependent impact of ZG-10 on viability of H9C2 cardiomyocytes. **(B)** Effect of low concentration ZG-10 on H9C2 cardiomyocytes viability after heat stress. **(C)** Heat stress-induced morphological changes in H9C2 cardiomyocytes. **(D, E)** TUNEL staining of apoptosis in H9C2 cardiomyocytes across each groups. Quantitative analysis of apoptotic cells, scale bar = 100 μm, n = 8. **(F–I)** The fluorescence images and relative fluorescence intensity of c-Jun and IL-1β in H9C2 cardiomyocytes, scale bar = 25 μm/100 μm, n = 5. **(J–O)** Representative western blot bands of c-Jun, cleaved-caspase 3, caspase 3, Bax and Bcl2 in H9C2 cardiomyocytes. Quantitative analysis of protein expression levels (normalized to β-actin), n = 5.

Furthermore, we validated the protective effects of ZG-10 on HS-induced myocardial injury. Initially, we pre-treated normal H9C2 cells with ZG-10 at different concentrations to establish its cytotoxicity. No effect was observed on cell viability at low ZG-10 concentrations (5, 10, and 20 μM), whereas cell viability decreased significantly in a dose-dependent manner at high ZG-10 concentrations (50 and 100 μM) (p < 0.001) (**Figure 9A**). Therefore, we selected the low ZG-10 concentrations (5, 10, and 20 μM) for the pre-treatment of H9C2 cells, followed by heat stress for 2 h and recovery at 37°C for 3 h. The results showed that pretreatment with 20 μM ZG-10 significantly improved cell viability (p < 0.05) and ameliorated heat stress-induced morphological changes in H9C2 cells (**Figures 9B–C**). Furthermore, TUNEL staining confirmed that ZG-10 significantly alleviated heat stress-induced cardiomyocyte apoptosis (**Figures 9D–E**). Immunofluorescence analysis showed that ZG-10 produced a pronounced reduction in the expression of c-Jun and IL-1β in heat stress-induced cardiomyocytes (**Figures 9F–I**). Western blotting results corroborated these findings and showed that ZG-10 inhibited c-Jun expression and significantly attenuated the expression of pro-apoptosis proteins such as cleaved-caspase 3 and Bax in HS-induced cardiomyocytes, whereas it upregulated the anti-apoptotic protein BCL2 (p < 0.05) (**Figures 9J–O**). These results confirmed that ZG-10 mitigates heat stress-induced cardiomyocyte injury by suppressing c-Jun-mediated apoptosis and inflammatory responses.

### ZG-10 ameliorates HS-induced myocardial injury by modulating JNK/p38 MAPK pathway

**Figure 10:**
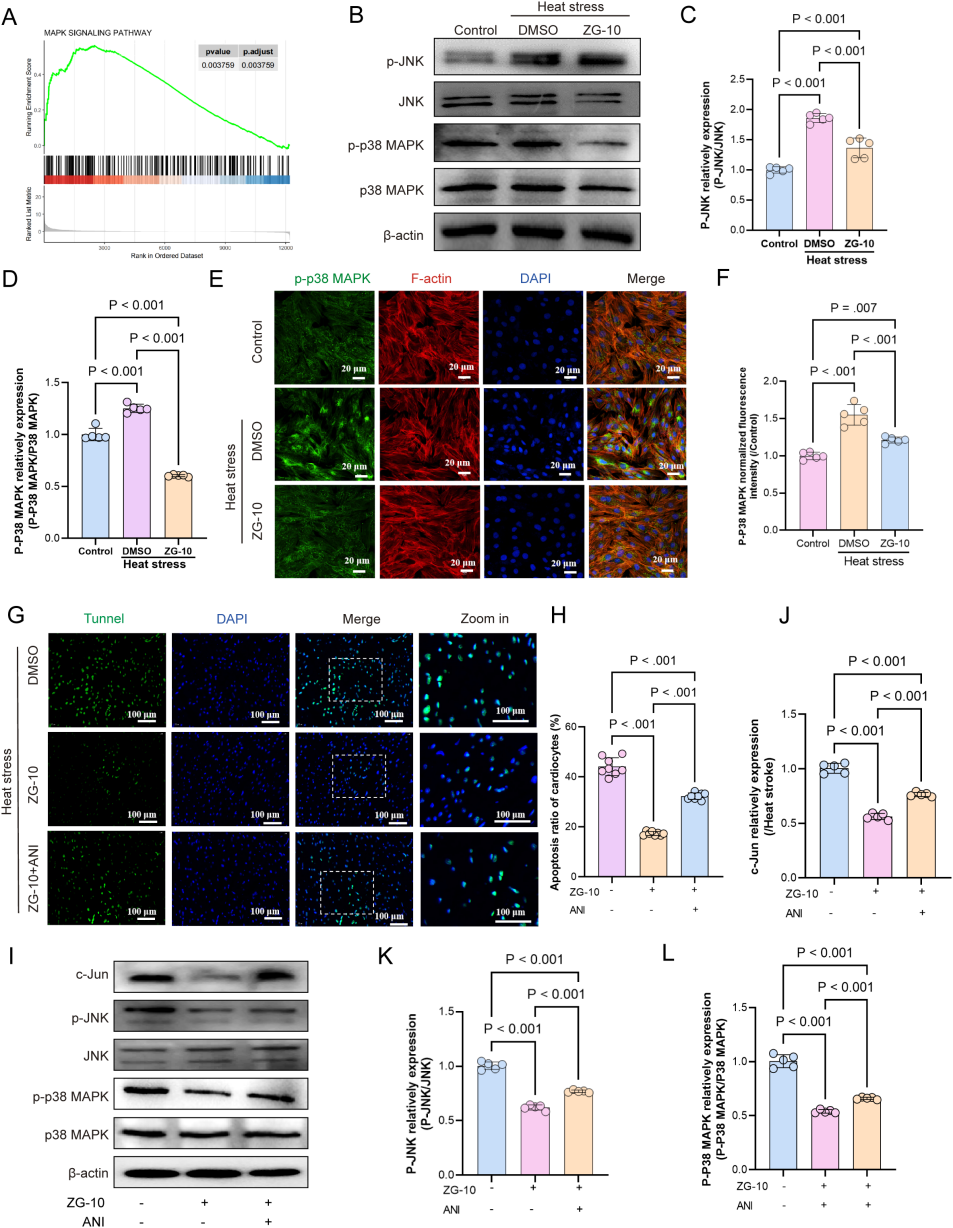
Mechanistic validation of ZG-10 in alleviating HS-induced myocardial injury. **(A)** GSEA analysis of MAPK signaling pathway. **(B–D)** Representative western blot bands of p-JNK, JNK, p-p38 MAPK and p38 MAPK in H9C2 cardiomyocytes. Quantitative analysis of protein expression levels (normalized to β-actin), n = 5. **(E, F)** The fluorescence images and relative fluorescence intensity of p-p38 MAPK in H9C2 cardiomyocytes, scale bar = 20 μm, n = 5. **(G, H)** TUNEL staining of H9C2 cardiomyocyte apoptosis across each groups. Quantitative analysis of apoptotic cells, scale bar = 100 μm, n = 8. **(I–L)** Representative western blot bands of c-Jun, p-JNK, JNK, p-p38 MAPK and p38 MAPK in myocardial tissue. Quantitative analysis of protein expression levels (normalized to β-actin), n = 5.

KEGG enrichment analysis showed significant enrichment of the DEGs associated with HS-induced myocardial injury in inflammatory, TNF, and MAPK signaling pathways. GSEA showed substantial upregulation of the MAPK signaling pathway in HS-induced myocardial tissues (**Figure 10A**). In mammals, the MAPK family can be divided into three main subfamilies: extracellular signal-regulated kinases (ERK), c-Jun N-terminal kinases (JNK), and p38/stress-activated protein kinases (p38/SAPKs) (47). Different MAPK subfamilies are involved in distinct signaling pathways with specific functions. For instance, the ERK pathway primarily regulates cell growth and differentiation (48). In contrast, both the JNK and p38 MAPK pathways play crucial roles in stress responses, such as inflammation and apoptosis (49, 50). Therefore, we hypothesize that ZG-10 alleviates HS-induced myocardial injury by suppressing apoptosis and inflammation through the inhibition of the JNK/p38 MAPK signaling pathway. Western blotting and immunofluorescence analyses confirmed JNK and p38 activation in heat stress-induced cardiomyocytes, whereas ZG-10 significantly reduced their phosphorylation (p < 0.001) (**Figures 10B–F**). Next, we validated the indispensable role of the JNK/p38 MAPK pathway in mediating the therapeutic effects of ZG-10 on HS-induced myocardial injury using the pharmacological JNK and p38 MAPK pathway activator Anisomycin (ANI) (51–53). TUNEL staining results showed that ANI partially abolished the therapeutic effects of ZG-10 against heat stress-induced cardiomyocyte injury (**Figures 10G–H**). We further introduced ANI into the heat stroke mouse model treated with ZG-10. Western blotting analysis confirmed that ANI counteracted ZG-10-mediated inhibition of c-Jun expression by reactivating JNK and p38 MAPK signaling (p < 0.001) (**Figures 10I–L**). Collectively, these findings confirmed that ZG-10 alleviates HS-induced myocardial injury through the suppression of the JNK/p38 MAPK signaling pathway.

## Discussion

Myocardial injury occurs as early as day 1 of HS with significant increase in serum cTnI levels (54). Heat injury leads to myocardial cell degeneration and necrosis, myocardial structure alteration, and cardiac conduction system and circulatory function damage (55). Most patients with HS initially exhibit a typical hyperdynamic hemodynamic state; however, as the disease progresses, stroke volume and maximal oxygen uptake gradually decrease (56). Moreover, HS-induced myocardial damage is not limited to acute phases, and patients exhibit signs of myocardial edema and fibrosis on cardiac magnetic resonance imaging (57). Furthermore, patients with HS complicated by myocardial injury often show poor prognosis and face higher treatment costs (58). Therefore, timely identification and intervention for management of myocardial injury in HS are critical.

In this study, WGCNA and transcriptome sequencing of HS-induced myocardial tissue helped identify 13 candidate DEGs. Liu et al. (59) characterized inflammation- and oxidative stress-related genes in a HS model and proposed that immune cells such as macrophages are critical determinants of HS prognosis. Therefore, we performed immune infiltration analysis on the candidate DEGs and found that the HS group was enriched in monocytes, macrophages, and other immune cells. HS is frequently accompanied by a massive release of inflammatory factors (60). Activated monocytes and macrophages are the central drivers of the cytokine storm and produce large quantities of pro-inflammatory cytokines including TNF-α, IL-1β, and IL-6. Furthermore, these cytokines propagate the inflammatory response, which ultimately leads to systemic inflammatory response syndrome (SIRS) (36, 61). The candidate DEGs exhibited negative correlation with Tr1 cells, CD8^+^ T cells, exhausted T cells, and cytotoxic T cells. T cells exert a suppressive effect on inflammatory cytokine production from innate immune cells during HS. The T-cell depletion observed in the HS mouse models indicate higher mortality and exacerbated inflammation, which could be rescued by T-cell transfer for mitigating disease severity (37). This is similar to the role of T cells in endotoxemia and sepsis (62, 63). These findings suggest that the candidate DEGs are key regulatory genes involved in the inflammatory response during HS. To identify the hub genes associated with HS-induced myocardial injury, we performed PPI analysis on the candidate DEGs. The results showed that JUN occupied a central position in the PPI network (with the highest degree value), which indicates its potential pivotal role in the pathogenesis and progression of HS-induced myocardial injury.

JUN encodes the c-Jun transcription factor, which primarily functions by forming homodimers or heterodimers with other transcription factors to bind to DNA and regulate gene expression. It plays an indispensable role in cellular processes such as proliferation, apoptosis, survival, and tissue morphogenesis (19, 43, 64). JNK-mediated phosphorylation of c-Jun activates downstream signaling pathways, and inhibition of the JNK/c-Jun axis alleviates myocardial injury induced by ischemia-reperfusion, doxorubicin, and sepsis (28, 29, 65). In this study, we collected blood samples from 80 patients with HS and found that the serum c-Jun levels were significantly higher in patients with HS-induced myocardial injury compared with the levels in those without. Furthermore, c-Jun levels positively correlated with cTnI levels with an AUC of 0.781 for diagnosing HS-induced myocardial injury. This indicates a strong association between c-Jun and severity of myocardial damage. Currently, few studies have explored diagnostic biomarkers for heat stroke-induced myocardial injury. Animal studies have identified a positive correlation between Hsp-72 levels and extent of myocardial damage, although this finding is yet to be validated in human samples (66). A retrospective clinical study has shown a U-shaped relationship between the triglyceride-glucose (TyG) index and myocardial injury in patients with HS with a decreasing trend in risk observed at TyG values ≤ 8.897 and pronounced increase in risk at values ≥ 8.966 (67). We emphasize that c-Jun activation, as an upstream signaling event, may confer an early diagnostic window prior to substantial cell death, potentially enabling earlier intervention. Notably, serum c-Jun levels were significantly high in patients who died within 28 days compared with that of the survivors. Based on these findings, we have developed a prognostic nomogram by integrating c-Jun levels, myocardial injury status, and SOFA scores. The model demonstrated excellent predictive accuracy with an AUC of 0.906, which highlights its clinical utility in forecasting HS outcomes.

Currently, prognostic biomarkers for patients with HS predominantly focus on hematological, biochemical, and clinical parameters such as WBC count, PLT count, comorbidities, body temperature, heart rate, and GCS score (68–71). To our knowledge, this study is the first to identify c-Jun as both a diagnostic and prognostic biomarker for HS-induced myocardial injury. Although we evaluated the performance of the model using DCA and calibration curves, which confirmed its clinical applicability and accuracy, several limitations remain. First, the clinical samples were sourced from a single center; therefore, they lacked external validation. Second, the sample size was limited. Nevertheless, the use of c-Jun as a biomarker via blood-based ELISA, which is a readily accessible method, warrants further investigation and broader application.

Direct thermal injury and secondary septic reactions occur in HS, owing to which patients with HS often develop severe systemic inflammatory responses that are characterized by excessive release of pro-inflammatory cytokines, activation of immune system, and subsequent infiltration of inflammatory cells, which lead to multi-organ damage (60, 72, 73). Hence, suppressing inflammatory responses is a critical strategy for treating HS-induced myocardial injury. Lin et al. (74) demonstrated have shown that myricetin may prevent HS-induced myocardial injury by alleviating oxidative stress and inflammation. Chen et al. (35) that inhibiting TLR4 could reduce inflammation and ferroptosis, thereby mitigating HS-induced myocardial injury. Furthermore, the JNK/c-Jun signaling pathway is closely associated with inflammatory responses and apoptosis revealed (75–77). Additionally, the current study has shown that heat stress significantly upregulates c-Jun expression in myocardial tissues and cells, accompanied by the activation of c-Jun-mediated inflammatory factors and apoptosis. Based on these findings, we screened potential therapeutic small molecules using the L1000 FWD database and identified ZG-10 as a potential therapeutic drug. Further *in vivo* and *in vitro* experiments showed that ZG-10 inhibited c-Jun-mediated inflammatory factors and apoptosis, which ameliorated HS-induced myocardial cell injury and cardiac dysfunction. Currently, no therapeutic agents have been explicitly recommended for myocardial injury-induced HS in clinical practice. However, similar to that observed during sepsis, heat stroke is often accompanied by a systemic inflammatory storm. This implies that corticosteroids may inhibit the release of pro-inflammatory factors (such as TNF-α and IL-6) and upregulate the expression of the anti-inflammatory factor IL-10, which would balance the excessive immune activation in the early stage of septic myocardial injury (78). Additionally, treatment with corticosteroids would regulate mitochondrial function in cardiomyocytes, reduce oxidative stress damage, and improve myocardial contractility (79). Nevertheless, high doses of corticosteroids may increase the risk of infection (80). Ulinastatin is a broad-spectrum anti-inflammatory agent that mitigates myocardial inflammation and microvascular endothelial injury (81), as evidenced by the reduction in the extent of myocardial damage in patients with myocardial injury after treatment with ulinastatin (82). However, caution is warranted because of its potential side effect of severe leukopenia (83). In contrast, JNK inhibitors are well tolerated by patients even after long-term use (24 weeks) with the most common adverse effects being diarrhea, nausea, and vomiting (84).

ZG-10 is a covalent JNK inhibitor that simultaneously targets JNK1, JNK2, and JNK3 with IC50 values of 809, 1140, and 709 nM, respectively (42, 43). In this study, KEGG enrichment and GSEA analyses showed significant enrichment of the MAPK signaling pathway. The JNK signaling pathway is a member of the MAPK family and is activated through a MAPK cascade, which leads to the translocation of JNK from the cytoplasm to the nucleus. This process results in the phosphorylation of the serine residues (Ser63 and Ser73) in the amino-terminal transactivation domain of c-Jun (85). Activated c-Jun binds to DNA and initiates the transcription of apoptosis-related genes by modulating the promoter activity of these genes and ultimately triggering cell apoptosis (47). p38 is one of the three major subunits of the MAPK family. p38 participates in a wide range of complex biological processes including cell proliferation, apoptosis, and differentiation (86, 87). The JNK and p38 signaling pathways jointly regulate the mitochondrial apoptotic pathway by altering the expression of specific pro-apoptotic and anti-apoptotic targets. Thus, they contribute to apoptotic response (88–90). Inhibiting the JNK/p38 MAPK signaling pathways may alleviate apoptosis in myocardial injury caused by ischemia-reperfusion (91, 92). These findings concur with our inference that ZG-10 significantly suppressed JNK and p38 MAPK phosphorylation in both *in vivo* and *in vitro* experiments under HS conditions. Collectively, our results show that ZG-10 ameliorates HS-induced myocardial inflammation and apoptosis by inhibiting JNK/p38 MAPK pathway activity and downregulating c-Jun expression.

Thus, this study has systematically elucidated the central regulatory role of c-Jun in HS-induced myocardial injury through integrated bioinformatics analysis, clinical sample validation, and *in vivo*/*in vitro* experiments. Finally, we constructed a prognostic model based on c-Jun and identified ZG-10 as a potential therapeutic agent for HS-induced myocardial injury. Nevertheless, this approach has certain limitations. First, the predictive model was developed using a limited clinical sample-size of single-center origin, which necessitates validating using a large sample-size of multicenter origin. Second, although ZG-10 improved myocardial injury in HS mice, its long-term effects on cardiovascular sequelae in human HS survivors remain unclear. Third, although ZG-10 inhibited the JNK/p38 MAPK pathway and downregulated c-Jun expression, the specific molecular targets (e.g., JNK isoform selectivity) and downstream effector molecules remain unclear. Future studies are warranted for identifying these components. Finally, the use of a mice model, which may exhibit species-specific differences in heat-stress responses compared with those of humans, necessitates validation using non-human primate models.

## Conclusion

In this study, we have systematically elucidated the central role of c-Jun in HS-induced myocardial injury. Consequently, we have developed a prognostic model based on c-Jun and identified ZG-10 as a potential therapeutic agent for HS-induced myocardial injury. ZG-10 exerts protective effects by inhibiting the JNK/p38 MAPK pathway, downregulating c-Jun expression, and effectively alleviating myocardial inflammation and apoptosis while improving cardiac function. These findings not only provide novel biomarkers for the early diagnosis and prognosis of HS-induced myocardial injury but also lay a theoretical foundation for the development of targeted therapeutic drugs. Future studies should focus on advancing clinical translation to improve survival and long-term outcomes in patients with HS.

## Data Availability

The datasets presented in this study can be found in online repositories. The names of the repository/repositories and accession number(s) can be found below: GSE303366 (GEO).

## References

[1] Song F Dong H Wu L Leung LR Lu J Dong L . Hot season gets hotter due to rainfall delay over tropical land in a warming climate. Nat Commun. (2025) 16:2188. doi: 10.1038/s41467-025-57501-6, PMID: 40038271 PMC11880391

[2] Wang Y Zhao N Yin X Wu C Chen M Jiao Y . Global future population exposure to heatwaves. Environ Int. (2023) 178:108049. doi: 10.1016/j.envint.2023.108049, PMID: 37379721

[3] Gupta K Khuttan A Kakar TS . Heatstroke. New Engl J Med. (2019) 381:1186. doi: 10.1056/NEJMc1909690, PMID: 31532981

[4] Bouchama A Abuyassin B Lehe C Laitano O Jay O O’Connor FG . Classic and exertional heatstroke. Nat Rev Dis Primers. (2022) 8:8. doi: 10.1038/s41572-021-00334-6, PMID: 35115565

[5] Al-Kindi S Motairek I Khraishah H Rajagopalan S . Cardiovascular disease burden attributable to non-optimal temperature: analysis of the 1990–2019 global burden of disease. Eur J Prev Cardiol. (2023) 30:1623–31. doi: 10.1093/eurjpc/zwad130, PMID: 37115593

[6] Xia R Sun M Li Y Yin J Liu H Yang J . The pathogenesis and therapeutic strategies of heat stroke-induced myocardial injury. Front Pharmacol. (2023) 14:1286556. doi: 10.3389/fphar.2023.1286556, PMID: 38259273 PMC10800451

[7] Yezli S Yassin Y Ghallab S Abdullah M Abuyassin B Vishwakarma R . Classic heat stroke in a desert climate: A systematic review of 2632 cases. J Intern Med. (2023) 294:7–20. doi: 10.1111/joim.13633, PMID: 36951097

[8] Misset B De Jonghe B Bastuji-Garin S Gattolliat O Boughrara E Annane D . Mortality of patients with heatstroke admitted to intensive care units during the 2003 heat wave in France: a national multiple-center risk-factor study. Crit Care Med. (2006) 34:1087–92. doi: 10.1097/01.CCM.0000206469.33615.02, PMID: 16484920

[9] Argaud L Ferry T Le QH Marfisi A Ciorba D Achache P . Short- and long-term outcomes of heatstroke following the 2003 heat wave in Lyon, France. Arch Intern Med. (2007) 167:2177–83. doi: 10.1001/archinte.167.20.ioi70147, PMID: 17698677

[10] Leon LR Bouchama A . Heat stroke. Compr Physiol. (2015) 5:611–47. doi: 10.1002/j.2040-4603.2015.tb00612.x 25880507

[11] Bouchama A Dehbi M Mohamed G Matthies F Shoukri M Menne B . Prognostic factors in heat wave related deaths: a meta-analysis. Arch Intern Med. (2007) 167:2170–6. doi: 10.1001/archinte.167.20.ira70009, PMID: 17698676

[12] Wang JC Chien WC Chu P Chung CH Lin CY Tsai SH . The association between heat stroke and subsequent cardiovascular diseases. PloS One. (2019) 14:e0211386. doi: 10.1371/journal.pone.0211386, PMID: 30759128 PMC6373898

[13] Tek D Olshaker JS . Heat illness. Emerg Med Clin North Am. (1992) 10:299–310. doi: 10.1016/S0733-8627(20)30714-8 1559470

[14] Epstein Y Yanovich R . Heatstroke. New Engl J Med. (2019) 380:2449–59. doi: 10.1056/NEJMra1810762, PMID: 31216400

[15] Roberts GT Ghebeh H Chishti MA Al-Mohanna F El-Sayed R Al-Mohanna F . Microvascular injury, thrombosis, inflammation, and apoptosis in the pathogenesis of heatstroke: a study in baboon model. Arterioscler Thromb Vasc Biol. (2008) 28:1130–6. doi: 10.1161/ATVBAHA.107.158709, PMID: 18388331

[16] Shen HH Tseng YS Kuo NC Kung CW Amin S Lam KK . Alpha-lipoic acid protects cardiomyocytes against heat stroke-induced apoptosis and inflammatory responses associated with the induction of hsp70 and activation of autophagy. Mediators Inflammation. (2019) 2019:8187529. doi: 10.1155/2019/8187529, PMID: 31885498 PMC6914879

[17] Yu M Wen N Wenzhong Z Yuanchang X Xiaoming D Yongjin L . Effect of repeated ischaemic preconditioning on TLR4 and proinflammatory cytokines TNF-α and IL-1β in myocardial ischaemia-reperfusion injury in a rat model. Arch Med Sci. (2010) 6:843–7. doi: 10.5114/aoms.2010.19289, PMID: 22427755 PMC3302693

[18] Angel P Karin M . The role of Jun, Fos and the AP-1 complex in cell-proliferation and transformation. Biochim Biophys Acta. (1991) 1072:129–57. doi: 10.1016/0304-419X(91)90011-9, PMID: 1751545

[19] Meng Q Xia Y . c-Jun, at the crossroad of the signaling network. Protein Cell. (2011) 2:889–98. doi: 10.1007/s13238-011-1113-3, PMID: 22180088 PMC4875184

[20] Miyake T McDermott JC . Nucleolar localization of c-Jun. FEBS J. (2022) 289:748–65. doi: 10.1111/febs.16187, PMID: 34499807

[21] Schonthaler HB Guinea-Viniegra J Wagner EF . Targeting inflammation by modulating the Jun/AP-1 pathway. Ann Rheumatol Dis. (2011) 70 Suppl 1:i109–12. doi: 10.1136/ard.2010.140533, PMID: 21339212

[22] Shvedova M Anfinogenova Y AtoChina-Vasserman EN Schepetkin IA Atochin DN . c-jun N-terminal kinases (JNKs) in myocardial and cerebral ischemia/reperfusion injury. Front Pharmacol. (2018) 9:715. doi: 10.3389/fphar.2018.00715, PMID: 30026697 PMC6041399

[23] Yang F Li M Xu D Jiang Z Jiang H Xiao Y . Inhibition of JNK/c-Jun-ATF2 Overcomes Cisplatin Resistance in Liver Cancer through down-Regulating Galectin-1. Int J Biol Sci. (2023) 19:2366–81. doi: 10.7150/ijbs.79163, PMID: 37215991 PMC10197891

[24] Zhang Q Hui M Chen G Huang H Wang S Ye Y . Curcumin-piperlongumine hybrid molecule increases cell cycle arrest and apoptosis in lung cancer through JNK/c-jun signaling pathway. J Agric Food Chem. (2024) 72:7244–55. doi: 10.1021/acs.jafc.4c00882, PMID: 38517372

[25] Kumar V Sabaté-Cadenas X Soni I Stern E Vias C Ginsberg D . The lincRNA JUNI regulates the stress-dependent induction of c-Jun, cellular migration and survival through the modulation of the DUSP14-JNK axis. Oncogene. (2024) 43:1608–19. doi: 10.1038/s41388-024-03021-4, PMID: 38565943 PMC11108773

[26] Chang HY Hsu HC Fang YH Liu PY Liu YW . Empagliflozin attenuates doxorubicin-induced cardiotoxicity by inhibiting the JNK signaling pathway. Biomed Pharmacother. (2024) 176:116759. doi: 10.1016/j.biopha.2024.116759, PMID: 38788603

[27] Xue HM Sun WT Chen HX He GW Yang Q . Targeting IRE1α-JNK-c-jun/AP-1-sEH signaling pathway improves myocardial and coronary endothelial function following global myocardial ischemia/reperfusion. Int J Med Sci. (2022) 19:1460–72. doi: 10.7150/ijms.74533, PMID: 36035373 PMC9413556

[28] Zou R Shi W Chang X Zhang M Tan S Li R . The DNA-dependent protein kinase catalytic subunit exacerbates endotoxemia-induced myocardial microvascular injury by disrupting the MOTS-c/JNK pathway and inducing profilin-mediated lamellipodia degradation. Theranostics. (2024) 14:1561–82. doi: 10.7150/thno.92650, PMID: 38389837 PMC10879869

[29] Shu G Chen K Li J Liu B Chen X Wang J . Galangin alleviated Doxorubicin-induced cardiotoxicity by inhibiting ferroptosis through GSTP1/JNK pathway. Phytomedicine: Int J phytotherapy phytopharmacology. (2024) 134:155989. doi: 10.1016/j.phymed.2024.155989, PMID: 39217656

[30] Langfelder P Horvath S . WGCNA: an R package for weighted correlation network analysis. BMC Bioinf. (2008) 9:559. doi: 10.1186/1471-2105-9-559, PMID: 19114008 PMC2631488

[31] Miao YR Zhang Q Lei Q Luo M Xie GY Wang H . ImmuCellAI: A unique method for comprehensive T-cell subsets abundance prediction and its application in cancer immunotherapy. Advanced Sci (Weinheim Baden-Wurttemberg Germany). (2020) 7:1902880. doi: 10.1002/advs.201902880, PMID: 32274301 PMC7141005

[32] Liu SY Song JC Mao HD Zhao JB Song Q . Expert consensus on the diagnosis and treatment of heat stroke in China. Military Med Res. (2020) 7:1. doi: 10.1186/s40779-019-0229-2, PMID: 31928528 PMC6956553

[33] Thygesen K Alpert JS Jaffe AS Chaitman BR Bax JJ Morrow DA . Fourth universal definition of myocardial infarction (2018). Circulation. (2018) 138:e618–51. doi: 10.1161/CIR.0000000000000617, PMID: 30571511

[34] Wang Z Lachmann A Keenan AB Ma’ayan A . L1000FWD: fireworks visualization of drug-induced transcriptomic signatures. Bioinf (Oxford England). (2018) 34:2150–2. doi: 10.1093/bioinformatics/bty060, PMID: 29420694 PMC6454499

[35] Chen D Geng Y Deng Z Li P Xue S Xu T . Inhibition of TLR4 alleviates heat stroke-induced cardiomyocyte injury by down-regulating inflammation and ferroptosis. Molecules (Basel Switzerland). (2023) 28:1–16. doi: 10.3390/molecules28052297, PMID: 36903542 PMC10005438

[36] Lim CL Mackinnon LT . The roles of exercise-induced immune system disturbances in the pathology of heat stroke: the dual pathway model of heat stroke. Sports Med (Auckland N.Z.). (2006) 36:39–64. doi: 10.2165/00007256-200636010-00004, PMID: 16445310

[37] Tan Y Liu X Yu X Shen T Wang Z Luo Z . Lack of lymphocytes exacerbate heat stroke severity in male mice through enhanced inflammatory response. Int Immunopharmacol. (2021) 101:108206. doi: 10.1016/j.intimp.2021.108206, PMID: 34626875

[38] Cantet JM Yu Z Ríus AG . Heat stress-mediated activation of immune-inflammatory pathways. Antibiotics (Basel Switzerland). (2021) 10:1–20. doi: 10.3390/antibiotics10111285, PMID: 34827223 PMC8615052

[39] Au KM Min Y Tian X Zhang L Perello V Caster JM . Improving cancer chemoradiotherapy treatment by dual controlled release of wortmannin and docetaxel in polymeric nanoparticles. ACS nano. (2015) 9:8976–96. doi: 10.1021/acsnano.5b02913, PMID: 26267360 PMC4990743

[40] Evison BJ Sleebs BE Watson KG Phillips DR Cutts SM . Mitoxantrone, more than just another topoisomerase II poison. Medicinal Res Rev. (2016) 36:248–99. doi: 10.1002/med.21364, PMID: 26286294

[41] Cortes J Feldman E Yee K Rizzieri D Advani AS Charman A . Two dosing regimens of tosedostat in elderly patients with relapsed or refractory acute myeloid leukaemia (OPAL): a randomised open-label phase 2 study. Lancet Oncol. (2013) 14:354–62. doi: 10.1016/S1470-2045(13)70037-8, PMID: 23453583 PMC5557006

[42] Zhang T Inesta-Vaquera F Niepel M Zhang J Ficarro SB Machleidt T . Discovery of potent and selective covalent inhibitors of JNK. Chem Biol. (2012) 19:140–54. doi: 10.1016/j.chembiol.2011.11.010, PMID: 22284361 PMC3270411

[43] Zhu Y Shuai W Zhao M Pan X Pei J Wu Y . Unraveling the design and discovery of c-jun N-terminal kinase inhibitors and their therapeutic potential in human diseases. J medicinal Chem. (2022) 65:3758–75. doi: 10.1021/acs.jmedchem.1c01947, PMID: 35200035

[44] Weston CR Davis RJ . The JNK signal transduction pathway. Curr Opin Cell Biol. (2007) 19:142–9. doi: 10.1016/j.ceb.2007.02.001, PMID: 17303404

[45] Wagner EF Nebreda AR . Signal integration by JNK and p38 MAPK pathways in cancer development. Nat Rev Cancer. (2009) 9:537–49. doi: 10.1038/nrc2694, PMID: 19629069

[46] Win S Than TA Kaplowitz N . Mitochondrial P-JNK target, SAB (SH3BP5), in regulation of cell death. Front Cell Dev Biol. (2024) 12:1359152. doi: 10.3389/fcell.2024.1359152, PMID: 38559813 PMC10978662

[47] Morrison DK . MAP kinase pathways. Cold Spring Harbor Perspect Biol. (2012) 4:1–5. doi: 10.1101/cshperspect.a011254, PMID: 23125017 PMC3536342

[48] Yuan J Dong X Yap J Hu J The MAPK . and AMPK signalings: interplay and implication in targeted cancer therapy. J Hematol Oncol. (2020) 13:113. doi: 10.1186/s13045-020-00949-4, PMID: 32807225 PMC7433213

[49] Yang Z Zhang H Yin M Cheng Z Jiang P Feng M . Neurotrophin3 promotes hepatocellular carcinoma apoptosis through the JNK and P38 MAPK pathways. Int J Biol Sci. (2022) 18:5963–77. doi: 10.7150/ijbs.72982, PMID: 36263167 PMC9576519

[50] Yue J López JM . Understanding MAPK signaling pathways in apoptosis. Int J Mol Sci. (2020) 21:1–22. doi: 10.3390/ijms21072346, PMID: 32231094 PMC7177758

[51] Rosser EM Morton S Ashton KS Cohen P Hulme AN . Synthetic anisomycin analogues activating the JNK/SAPK1 and p38/SAPK2 pathways. Org Biomol Chem. (2004) 2:142–9. doi: 10.1039/b311242j, PMID: 14737674

[52] Nikaido M Otani T Kitagawa N Ogata K Iida H Anan H . Anisomycin, a JNK and p38 activator, suppresses cell-cell junction formation in 2D cultures of K38 mouse keratinocyte cells and reduces claudin-7 expression, with an increase of paracellular permeability in 3D cultures. Histochem Cell Biol. (2019) 151:369–84. doi: 10.1007/s00418-018-1736-z, PMID: 30284609

[53] Chung KC Kim SM Rhang S Lau LF Gomes I Ahn YS . Expression of immediate early gene pip92 during anisomycin-induced cell death is mediated by the JNK- and p38-dependent activation of Elk1. Eur J Biochem. (2000) 267:4676–84. doi: 10.1046/j.1432-1327.2000.01517.x, PMID: 10903500

[54] Dervišević E Hasić S Katica M Dervišević L Ajanović Z Salihbegović A . Heat-related biomarkers: Focus on the correlation of troponin I and 70 kDa heat shock protein. Heliyon. (2023) 9:e14565. doi: 10.1016/j.heliyon.2023.e14565, PMID: 37025834 PMC10070379

[55] Chen WT Lin CH Hsieh MH Huang CY Yeh JS . Stress-induced cardiomyopathy caused by heat stroke. Ann Emergency Med. (2012) 60:63–6. doi: 10.1016/j.annemergmed.2011.11.005, PMID: 22153997

[56] Mulholland AM Yoder HA Wingo JE . Effect of work-to-rest cycles on cardiovascular strain and maximal oxygen uptake during heat stress. Int J Environ Res Public Health. (2023) 20:1–13. doi: 10.3390/ijerph20054580, PMID: 36901590 PMC10001546

[57] Luo S Xu ST Zhang J Schoepf UJ Varga-Szemes A Carpenter CRT . Multiparametric cardiac magnetic resonance reveals persistent myocardial inflammation in patients with exertional heat illness. Eur Radiol. (2023) 33:8165–76. doi: 10.1007/s00330-023-09706-w, PMID: 37145150

[58] Bathini T Thongprayoon C Chewcharat A Petnak T Cheungpasitporn W Boonpheng B . Acute myocardial infarction among hospitalizations for heat stroke in the United States. J Clin Med. (2020) 9:1–9. doi: 10.3390/jcm9051357, PMID: 32384601 PMC7290741

[59] Liu Z Chen J Hu L Li M Liang M Chen J . Expression profiles of genes associated with inflammatory responses and oxidative stress in lung after heat stroke. Bioscience Rep. (2020) 40:1–15. doi: 10.1042/BSR20192048, PMID: 32436952 PMC7276522

[60] Garcia CK Renteria LI Leite-Santos G Leon LR Laitano O . Exertional heat stroke: pathophysiology and risk factors. BMJ Med. (2022) 1:e000239. doi: 10.1136/bmjmed-2022-000239, PMID: 36936589 PMC9978764

[61] Yin D Guo Q Jiang H Hu Y Liu L Li X . Single-cell sequencing-based study of ferroptosis mechanisms in heat stroke: identification of key biomarkers and dynamic analysis of the immune microenvironment. BMC Med Genomics. (2025) 18:124. doi: 10.1186/s12920-025-02188-3, PMID: 40745616 PMC12315307

[62] Guarda G Dostert C Staehli F Cabalzar K Castillo R Tardivel A . T cells dampen innate immune responses through inhibition of NLRP1 and NLRP3 inflammasomes. Nature. (2009) 460:269–73. doi: 10.1038/nature08100, PMID: 19494813

[63] Kim KD Zhao J Auh S Yang X Du P Tang H . Adaptive immune cells temper initial innate responses. Nat Med. (2007) 13:1248–52. doi: 10.1038/nm1633, PMID: 17891146 PMC2435248

[64] Shi D Mu S Hu B Zhang S Liu J Zhang Z . Prognostic role of c-Jun activation domain-binding protein-1 in cancer: A systematic review and meta-analysis. J Cell Mol Med. (2021) 25:2750–63. doi: 10.1111/jcmm.16334, PMID: 33550701 PMC7957274

[65] Zeng JJ Shi HQ Ren FF Zhao XS Chen QY Wang DJ . Notoginsenoside R1 protects against myocardial ischemia/reperfusion injury in mice via suppressing TAK1-JNK/p38 signaling. Acta pharmacologica Sin. (2023) 44:1366–79. doi: 10.1038/s41401-023-01057-y, PMID: 36721009 PMC10310839

[66] Dehbi M Baturcam E Eldali A Ahmed M Kwaasi A Chishti MA . Hsp-72, a candidate prognostic indicator of heatstroke. Cell Stress chaperones. (2010) 15:593–603. doi: 10.1007/s12192-010-0172-3, PMID: 20174993 PMC3006628

[67] Tang G Yang Y Zhang T Cheng T Gao H Wei W . Association between triglyceride-glucose index and myocardial injury in patients with heat stroke: an observational, retrospective study. Sci Rep. (2025) 15:34595. doi: 10.1038/s41598-025-18128-1, PMID: 41044245 PMC12494901

[68] Zhong L Wu M Ji J Liu Z . Usefulness of sequential organ failure assessment score on admission to predict the 90-day mortality in patients with exertional heatstroke: An over 10-year intensive care survey. Am J Emergency Med. (2022) 61:56–60. doi: 10.1016/j.ajem.2022.08.042, PMID: 36049393

[69] Wang F Gong F Shi X Yang J Qian J Wan L . Monocyte HLA-DR level on admission predicting in-hospital mortality rate in exertional heatstroke: A 12-year retrospective study. Immunity Inflammation Dis. (2024) 12:e1240. doi: 10.1002/iid3.1240, PMID: 38629749 PMC11022625

[70] Shao F Shi X Huo SH Liu QY Shi JX Kang J . Development and evaluation of a predictive nomogram for survival in heat stroke patients: a retrospective cohort study. World J Emergency Med. (2022) 13:355–60. doi: 10.5847/wjem.j.1920-8642.2022.092, PMID: 36119776 PMC9420659

[71] Wang Y Li D Wu Z Zhong C Tang S Hu H . Development and validation of a prognostic model of survival for classic heatstroke patients: a multicenter study. Sci Rep. (2023) 13:19265. doi: 10.1038/s41598-023-46529-7, PMID: 37935703 PMC10630318

[72] Leon LR Helwig BG . Heat stroke: role of the systemic inflammatory response. J Appl Physiol (Bethesda Md.: 1985). (2010) 109:1980–8. doi: 10.1152/japplphysiol.00301.2010, PMID: 20522730

[73] Wang Z Zhu J Zhang D Lv J Wu L Liu Z . The significant mechanism and treatments of cell death in heatstroke. Apoptosis: an Int J programmed Cell Death. (2024) 29:967–80. doi: 10.1007/s10495-024-01979-w, PMID: 38886312

[74] Lin X Lin CH Liu R Li C Jiao S Yi X . Myricetin against myocardial injury in rat heat stroke model. Biomedicine pharmacotherapy = Biomedecine pharmacotherapie. (2020) 127:110194. doi: 10.1016/j.biopha.2020.110194, PMID: 32371315

[75] Guo M Härtlova A Gierliński M Prescott A Castellvi J Losa JH . Triggering MSR1 promotes JNK-mediated inflammation in IL-4-activated macrophages. EMBO J. (2019) 38:1–15. doi: 10.15252/embj.2018100299, PMID: 31028084 PMC6545745

[76] Feng L Ning R Liu J Liang S Xu Q Liu Y . Silica nanoparticles induce JNK-mediated inflammation and myocardial contractile dysfunction. J hazardous materials. (2020) 391:122206. doi: 10.1016/j.jhazmat.2020.122206, PMID: 32036317

[77] Zhang K Zhang MX Meng XX Zhu J Wang JJ He YF . Targeting GPR65 alleviates hepatic inflammation and fibrosis by suppressing the JNK and NF-κB pathways. Military Med Res. (2023) 10:56. doi: 10.1186/s40779-023-00494-4, PMID: 38001521 PMC10675918

[78] Venkatesh B Finfer S Cohen J Rajbhandari D Arabi Y Bellomo R . Adjunctive glucocorticoid therapy in patients with septic shock. New Engl J Med. (2018) 378:797–808. doi: 10.1056/NEJMoa1705835, PMID: 29347874

[79] Rog-Zielinska EA Craig MA Manning JR Richardson RV Gowans GJ Dunbar DR . Glucocorticoids promote structural and functional maturation of foetal cardiomyocytes: a role for PGC-1α. Cell Death differentiation. (2015) 22:1106–16. doi: 10.1038/cdd.2014.181, PMID: 25361084 PMC4572859

[80] Lv J Zhang H Wong MG Jardine MJ Hladunewich M Jha V . Effect of oral methylprednisolone on clinical outcomes in patients with igA nephropathy: the TESTING randomized clinical trial. Jama. (2017) 318:432–42. doi: 10.1001/jama.2017.9362, PMID: 28763548 PMC5817603

[81] Qiu J Xiao X Gao X Zhang Y . Ulinastatin protects against sepsis−induced myocardial injury by inhibiting NLRP3 inflammasome activation. Mol Med Rep. (2021) 24:1–9. doi: 10.3892/mmr.2021.12369, PMID: 34414461 PMC8404092

[82] Wang S Dong Y Wang J Leng L Song X Huang W . The protective effect of ulinastatin combined with Xuebijing on myocardial injuries in patients with severe pneumonia. Am J Trans Res. (2021) 13:11745–51., PMID: 34786102 PMC8581934

[83] Li J Li M Li L Ma L Cao A Wen A . Real-world safety of ulinastatin: a post-marketing surveillance of 11,252 patients in China. BMC Pharmacol Toxicol. (2022) 23:51. doi: 10.1186/s40360-022-00585-3, PMID: 35842685 PMC9288682

[84] Mattos W Khalil N Spencer LG Bonella F Folz RJ Rolf JD . Phase 2, double-blind, placebo-controlled trial of a c-jun N-terminal kinase inhibitor in idiopathic pulmonary fibrosis. Am J Respir Crit Care Med. (2024) 210:435–43. doi: 10.1164/rccm.202310-1907OC, PMID: 38484130

[85] Li L Zhang G Yang Z Kang X . Stress-activated protein kinases in intervertebral disc degeneration: unraveling the impact of JNK and p38 MAPK. Biomolecules. (2024) 14:1–28. doi: 10.3390/biom14040393, PMID: 38672411 PMC11047866

[86] Ganguly P Macleod T Wong C Harland M McGonagle D . Revisiting p38 mitogen-activated protein kinases (MAPK) in inflammatory arthritis: A narrative of the emergence of MAPK-activated protein kinase inhibitors (MK2i). Pharm (Basel Switzerland). (2023) 16:1–13. doi: 10.3390/ph16091286, PMID: 37765094 PMC10537904

[87] Sarg NH Zaher DM Abu Jayab NN Mostafa SH Ismail HH Omar HA . The interplay of p38 MAPK signaling and mitochondrial metabolism, a dynamic target in cancer and pathological contexts. Biochem Pharmacol. (2024) 225:116307. doi: 10.1016/j.bcp.2024.116307, PMID: 38797269

[88] Meng L Yuan L Ni J Fang M Guo S Cai H . Mir24-2-5p suppresses the osteogenic differentiation with Gnai3 inhibition presenting a direct target via inactivating JNK-p38 MAPK signaling axis. Int J Biol Sci. (2021) 17:4238–53. doi: 10.7150/ijbs.60536, PMID: 34803495 PMC8579458

[89] Chen X Ma W Yao Y Zhang Q Li J Wu X . Serum deprivation-response protein induces apoptosis in hepatocellular carcinoma through ASK1-JNK/p38 MAPK pathways. Cell Death Dis. (2021) 12:425. doi: 10.1038/s41419-021-03711-x, PMID: 33931585 PMC8087765

[90] Shen H Yuan J Tong D Chen B Yu E Chen G . Regulator of G protein signaling 16 restrains apoptosis in colorectal cancer through disrupting TRAF6-TAB2-TAK1-JNK/p38 MAPK signaling. Cell Death Dis. (2024) 15:438. doi: 10.1038/s41419-024-06803-6, PMID: 38906869 PMC11192724

[91] Liu T Zhou Y Liu YC Wang JY Su Q Tang ZL . Coronary microembolization induces cardiomyocyte apoptosis through the LOX-1-dependent endoplasmic reticulum stress pathway involving JNK/P38 MAPK. Can J Cardiol. (2015) 31:1272–81. doi: 10.1016/j.cjca.2015.01.013, PMID: 26095939

[92] Zhou W Cai D . Midazolam suppresses ischemia/reperfusion-induced cardiomyocyte apoptosis by inhibiting the JNK/p38 MAPK signaling pathway. Can J Physiol Pharmacol. (2022) 100:117–24. doi: 10.1139/cjpp-2021-0289, PMID: 34559975

