# Correction: Targeting c-Jun orchestrates heat stroke-induced myocardial injury and reveals its biomarker potential

**DOI:** 10.3389/fimmu.2026.1778549

**Published:** 2026-01-12

**Authors:** Yunfei Xiang, Rui Huang, Xuemei Jiang, Qingsong Chen, Can Luo, Hongxia Wang, Junyu Jiang, Jia Xie, Guangbin Huang, Menglong Liu, Yu Ma, Dingyuan Du, Fating Zhou

**Affiliations:** 1 Department of Traumatology, Chongqing Emergency Medical Center, Chongqing University Central Hospital, School of Medicine, Chongqing University, Chongqing, China; 2 Chongqing Key Laboratory of Emergency Medicine, Chongqing Emergency Medical Center, Chongqing University Central Hospital, School of Medicine, Chongqing University, Chongqing, China; 3 Department of Emergency Medicine, Chongqing Emergency Medical Center, Chongqing University Central Hospital, Chongqing, China; 4 Department of Emergency, Affiliated Hospital of Zunyi Medical University, Zunyi, Guizhou, China; 5 Department of Traumatology, Chongqing Emergency Medical Center, Chongqing University Central Hospital, Chongqing, China; 6 Department of General Practice, People’s Hospital of Deyang City, Deyang, Sichuan, China

**Keywords:** heat stroke, myocardial injury, c-Jun, ZG-10, JNK/p38MAPK signaling

Author Can Luo, Menglong Liu were erroneously assigned to the original affiliation 5 Department of Traumatology, Chongqing Emergency Medical Center, Chongqing University Central Hospital, Chongqing, China. The affiliation has now been correctly assigned to affiliation 4 Department of Emergency, Affiliated Hospital of Zunyi Medical University Zunyi, Guizhou, China for these author.

Author Junyu Jiang, Guangbin Huang, Dingyuan Du, Fating Zhou were erroneously assigned to the original affiliation 4 Department of Emergency, Affiliated Hospital of Zunyi Medical University Zunyi, Guizhou, China. The affiliation has now been correctly assigned to affiliation 5 Department of Traumatology, Chongqing Emergency Medical Center, Chongqing University Central Hospital, Chongqing, China for these author.

The original article has been updated.

