# Supplementary material for: Targeting c-Jun orchestrates heat stroke-induced myocardial injury and reveals its biomarker potential
Source: Front Immunol. 2025 Nov 12;16:1679750. doi: 10.3389/fimmu.2025.1679750 (PMC12646939; doi:10.3389/fimmu.2025.1679750)
Supplement: Supplementary file 1 [file Supplementaryfile1.docx]

**Supplementary Table1.** Primers used for qPCR

| **Gene Name** | **Forward Primer** | **Reverse Primer** |
| --- | --- | --- |
| JUN | CCTTCTACGACGATGCCCTC | GGTTCAAGGTCATGCTCTGTTT |
| IL-1β | GCAACTGTTCCTGAACTCAACT | ATCTTTTGGGGTCCGTCAACT |
| IL-6 | TAGTCCTTCCTACCCCAATTTCC | TTGGTCCTTAGCCACTCCTTC |
| TNF-α | GACGTGGAACTGGCAGAAGAG | TTGGTGGTTTGTGAGTGTGAG |
| β-actin | CATGTACGTTGCTATCCAGGC | CTCCTTAATGTCACGCACGAT |

**Supplementary Table2.** Univariate Logistic Regression Analysis for the 28d mortality of heat stroke in 80 Patients

| **Factor** | **Univariate analysis** | | **p-value** |
| --- | --- | --- | --- |
|  | **Odds ratio** | **95% CI** |  |
| c-Jun | 1.21 | 1.11-1.35 | <0.001 |
| Sex |  |  |  |
| Male | - | - | - |
| Female | 0.90 | 0.37-2.20 | 0.820 |
| Age | 0.98 | 0.95-1.01 | 0.127 |
| WBC | 1.03 | 0.97-1.11 | 0.337 |
| PLT | 1.00 | 0.99-1.00 | 0.209 |
| MONO | 1.27 | 0.72-2.37 | 0.424 |
| NEUT | 1.02 | 0.95-1.10 | 0.614 |
| Myocardial injury |  |  |  |
| Yes | - | - | - |
| No | 0.07 | 0.02-0.20 | p<0.001 |
| Sofa | 1.65 | 1.31-2.16 | p<0.001 |

**Abbreviations:** WBC, white blood cell; PLT, platelet; MONO, monocyte; NEUT, Neutrophils.
